# Neural Correlates of Motor Skill Learning Are Dependent on Both Age and Task Difficulty

**DOI:** 10.3389/fnagi.2021.643132

**Published:** 2021-03-22

**Authors:** Josje M. Bootsma, Simone R. Caljouw, Menno P. Veldman, Natasha M. Maurits, John C. Rothwell, Tibor Hortobágyi

**Affiliations:** ^1^Department of Human Movement Sciences, University Medical Center Groningen, University of Groningen, Groningen, Netherlands; ^2^Movement Control and Neuroplasticity Research Group, Department of Movement Science, KU Leuven, Leuven, Belgium; ^3^Leuven Brain Institute, KU Leuven, Leuven, Belgium; ^4^Department of Neurology, University Medical Center Groningen, University of Groningen, Groningen, Netherlands; ^5^Sobell Department of Motor Neuroscience and Movement Disorders, University College London (UCL) Institute of Neurology, London, United Kingdom

**Keywords:** aging, electroencephalography, motor learning, plasticity, spectral analysis, task difficulty

## Abstract

Although a general age-related decline in neural plasticity is evident, the effects of age on neural plasticity after motor practice are inconclusive. Inconsistencies in the literature may be related to between-study differences in task difficulty. Therefore, we aimed to determine the effects of age and task difficulty on motor learning and associated brain activity. We used task-related electroencephalography (EEG) power in the alpha (8–12 Hz) and beta (13–30 Hz) frequency bands to assess neural plasticity before, immediately after, and 24-h after practice of a mirror star tracing task at one of three difficulty levels in healthy younger (19–24 yr) and older (65–86 yr) adults. Results showed an age-related deterioration in motor performance that was more pronounced with increasing task difficulty and was accompanied by a more bilateral activity pattern for older vs. younger adults. Task difficulty affected motor skill retention and neural plasticity specifically in older adults. Older adults that practiced at the low or medium, but not the high, difficulty levels were able to maintain improvements in accuracy at retention and showed modulation of alpha TR-Power after practice. Together, these data indicate that both age and task difficulty affect motor learning, as well as the associated neural plasticity.

## Introduction

Unfavorable structural, functional, and biochemical changes in the nervous system affect the speed and accuracy of movements in older adults (Smith et al., 1999; Seidler et al., 2010). However, if and how old age affects the acquisition and retention of new motor skills is still under debate (Voelcker-Rehage, 2008; Coats et al., 2014). Motor practice can modify the structure and function of neural populations involved in motor skill acquisition and retention (Kolb et al., 2003; Pascual-Leone et al., 2005). Although a general age-related decline in such neural plasticity is evident (Reuter-Lorenz and Park, 2010; Pascual-Leone et al., 2011), the age-effects on neural plasticity after a period of motor practice are inconclusive (King et al., 2013; Cai et al., 2014). Understanding the effects of age on motor learning and practice-related plasticity is important to aid functional independence of older adults in increasingly aging societies and to develop novel rehabilitation practice schemes.

When executing the same motor task, older compared with younger adults show greater and more widespread brain activation (Berghuis et al., 2019; Larivière et al., 2019). Two main models have been proposed to characterize the altered age-related activation patterns: a decrease in asymmetry between the hemispheres [Hemispheric Asymmetry Reduction in Older Adults; HAROLD (Cabeza, 2002)] and a shift in activation from posterior to more anterior areas of the brain [Posterior to Anterior Shift in Aging; PASA (Davis et al., 2008)]. Both models are mostly explained as compensatory mechanisms to counteract age-dictated structural and functional changes and minimize the deterioration of motor skills (Seidler et al., 2010). The Compensation-Related Utilization of Neural Circuits Hypothesis (CRUNCH) from the cognitive literature explains age-related overactivation at low levels of cognitive demand as the recruitment of more neural resources to compensate for processing deficiencies (Reuter-Lorenz and Cappell, 2008). However, when cognitive demands increase, compensation strategies in older adults may not be sufficient to maintain young-like task performance. Specifically, as a ceiling level in terms of neural resources is reached, older adults' task performance may consequently deteriorate, causing an increased age-related decline in task performance with increasing task difficulty (Reuter-Lorenz and Cappell, 2008). Evidence showing more pronounced age-related deterioration in motor performance with increasing task difficulty (Smith et al., 1999; Bangert et al., 2010), as well as increased activation in younger adults when the difficulty of a motor task increases (Rietschel et al., 2012; Buetefisch et al., 2014), provide experimental support for the idea that the CRUNCH model is applicable in the motor domain, as well.

While the effects of task difficulty on motor performance are well-described (Fitts, 1954), the effects of task difficulty on motor skill acquisition and retention are less clear. Task difficulty can be defined as the level of challenge to execute a motor task within the current spatial and temporal constraints (Bootsma et al., 2020). When a task becomes increasingly difficult, heightened motor, and cognitive demands might act as a stimulus for motor skill learning until processing capacities are exceeded (Guadagnoli and Lee, 2004). Indeed, retention performance in younger adults was maximized after practice at the highest of four difficulty levels of a keypress sequence (Lee et al., 2016) and the second-highest of four difficulty levels of a postural control task (Akizuki and Ohashi, 2015). In contrast, there are also studies reporting no effects of task difficulty on motor learning in younger adults (Joseph et al., 2013; Ong et al., 2015) or increased learning after practice at lower compared to higher difficulty levels (Maxwell et al., 2001; Chiviacowsky and Harter, 2015). Data on the effects of task difficulty on motor learning in older adults are scarce. In one study, older adults improved more after practice on an easier compared to a more difficult version of a force tracking task (Onushko et al., 2014), which is in line with the CRUNCH model.

To the best of our knowledge, no study to date has systematically examined the effects of age and task difficulty on motor learning, even though such data could provide additional insights into practice-related neural plasticity. Oscillatory activity in the alpha and beta frequency bands has been proposed as a marker of neural plasticity (Jensen et al., 2005; Bavelier et al., 2010). Electroencephalographic (EEG) power in the alpha and beta frequencies is known to be dominant at rest and to be suppressed during movements (Pfurtscheller and Lopes da Silva, 1999). During motor task execution, task-related power is lower in older compared to younger adults, indicative of higher neural activity (Heinrichs-Graham and Wilson, 2016). If and how old age affects changes in task-related power during motor practice is, however, less well-understood. In young adults, decreased task-related power following motor practice has been interpreted as an indication of early neural plasticity (Boonstra et al., 2007; Nakano et al., 2013). Findings in older adults are inconclusive: similar (Espenhahn et al., 2019) as well as reduced (Mary et al., 2015) neural plasticity in comparison with younger adults has been reported after a single practice period. These inconsistencies in the literature may be related to differences in task difficulty between studies. However, if and how age affects the interaction between task difficulty and neural plasticity is unknown.

Therefore, the aim of the current study was to determine the effects of age and task difficulty on motor performance and task-related EEG power before, immediately after, and 24-h after acquisition of a mirror star tracing task. The overarching hypothesis was that both age and task difficulty would affect motor learning and practice-related plasticity. More specifically, we hypothesized an age-related deterioration in motor performance that would be more pronounced with increasing task difficulty. Furthermore, we expected no differences between younger and older adults after practice at a low difficulty level, but lower learning rates for older vs. younger adults after practice at higher difficulty levels. With regard to neural activity, we hypothesized that older compared to younger adults would show more bilateral activity already during motor performance at a low difficulty level and that there would be an increase in bilateral activity with increasing task difficulty in both age groups. Finally, we hypothesized smaller changes in task-related EEG power after the practice period in older compared to younger adults, indicative of an age-related deterioration in neural plasticity. Similar to the age-related deterioration in motor performance, we expected this effect to be more pronounced at higher difficulty levels.

## Materials and Methods

### Participants

Data from healthy, right-handed (Oldfield, 1971) younger adults (*N* = 36, age: 21.1 ± 1.3 yr, 16 males) were compared with previously reported data in healthy, right-handed, community-dwelling older adults (*N* = 36, age: 70.4 ± 4.1 yr, 20 males) (Bootsma et al., 2020). The sample size was based on an a priori power analysis performed in G^*^Power (version 3.1.9.2) with an alpha of 0.05 and statistical power of 0.8. We determined effect sizes based on behavioral data published previously (Bootsma et al., 2018). To ensure that the sample size in the present study would be sufficient to observe the smallest behavioral effects, we chose the lowest partial eta squared value from those analyses (${\text{η}}_{\text{p}}^{2}$ = 0.15) as input for the power analysis in the present study. Exclusion criteria were: movement restrictions in the right upper extremity, neurological conditions, and medications affecting neural functioning. In addition, older adults were screened to be cognitively [Mini-Mental State Examination score 29.2 ± 1.1 (Folstein et al., 1975)] and physically [Groningen Activity Restriction Scale score 18.4 ± 1.5 (Kempen et al., 1996)] well-functioning. Written informed consent was obtained before testing and the local ethics committee approved the study protocol, which was carried out in accordance with the Declaration of Helsinki.

### Experimental Design

**Figure 1:**
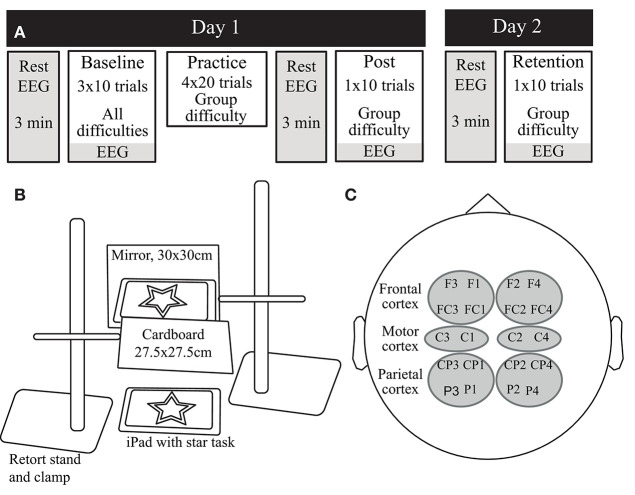
**(A)** The experimental design of the study. On day 1, participants practiced the visuomotor task at one of three difficulty levels. Before (baseline), immediately after (Post) and 24 h after (Retention) practice, motor performance, and EEG data were acquired. **(B)** The mirror star-tracing task. Participants were asked to trace the outline of a symmetrical five-point star as quickly and accurately as possible, while only allowed to look at the star and their moving hand through a mirror. **(C)** The combinations of electrodes used for the regions of interest in the EEG analysis.

Figure 1 shows the study design and visuomotor task, described in detail previously (Bootsma et al., 2020). Briefly, participants visited the lab on two consecutive days. On day 1, participants practiced a mirror star tracing task at one of three difficulty levels defined by the width of the wall of the star (i.e., the pathway): Practice with Low Difficulty (P-LD, 7 mm), Practice with Medium Difficulty (P-MD, 5 mm), or Practice with High Difficulty (P-HD, 3 mm). Participants were instructed to trace the outline of the star as quickly and accurately as possible with an Apple Pencil held in their right, dominant hand, while they could only see the star and their moving hand through a mirror. Before motor practice, participants performed ten trials on each difficulty level in a block-randomized order to assess baseline performance. Practice consisted of four blocks of twenty trials separated by 5 min rest periods. Immediately after and 24 h after (day 2) motor practice, resting-state EEG, motor performance on the practiced difficulty level, and task-related EEG were assessed. Motor performance was quantified in terms of both speed (movement time) and accuracy (bandwidth error). Movement time was the average time to complete one trial of the star task. Bandwidth error was the average percentage of samples in a trial that was outside of the wall of the star. At the end of day 1, perceived mental workload was measured with the NASA-tlx questionnaire (Hart and Staveland, 1988) and participants rated their perceived fatigue on a visual analog scale ranging from 0 (not fatigued at all) to 10 (completely fatigued).

### EEG Data Acquisition and Preprocessing

EEG signals were continuously recorded at 250 Hz in a shielded room using a 64-channel active electrode system placed according to the international 10–10 configuration (Brain Products GmbH, Germany, Chatrian et al., 1985). The ground electrode was placed between Fp1 and Fp2, Fz was used as a recording reference and the impedance of all electrodes was kept below 10 kΩ.

We preprocessed and analyzed the EEG data using the Fieldtrip Toolbox implemented in Matlab (Oostenveld et al., 2011), as detailed previously (Bootsma et al., 2020). Briefly, resting-state and task-related data were low-pass filtered (4^th^ order Butterworth, 70 Hz), band-stop filtered at 50 Hz, re-referenced with an average reference, segmented into non-overlapping 1-s-long epochs, upsampled to 256 Hz using piecewise cubic interpolation, and cleaned from eye-blinks and trials containing artifacts. The first and last 10% of the data of each trial were discarded before epoching to exclude errors related to the start and end of a trial. Power spectra were then calculated from the cleaned EEG data using a Fast Fourier Transform with a 10% Hanning window. Task-related power (TR-Power) spectra were averaged over the alpha (8–12 Hz) and beta (13–30 Hz) frequency bands and expressed as a percentage of power change during task execution relative to the resting-state power, according to the following equation:

(1) $$\begin{array}{r} TR-Power=\frac{Power_{task}-\;Power_{rest}}{Power_{rest}}\;\times 100 \end{array}$$

Subsequently, we calculated TR-Power for regions of interest (ROIs) over the contra- and ipsilateral frontal cortex (contralateral: average of F3, FC3, F1, and FC1; ipsilateral: average of F4, FC4, F2, and FC2), motor cortex (contralateral: average of C3 and C1; ipsilateral: average of C4 and C2) and parietal cortex (contralateral: average of CP3, P3, CP1, and P1; ipsilateral: average of CP4, P4, CP2, and P2).

### Statistical Analysis

All statistical analyses were conducted using R (R Core Team, 2020) in combination with the lme4 (Bates et al., 2015), lmerTest (Kuznetsova et al., 2017), and rstatix (Alboukadel, 2020) packages. Data were checked for normality with the Shapiro-Wilk test and log-transformed when the distribution was not normal. All results are reported untransformed (mean ± SD). From three younger and two older adults, EEG data at one of the three timepoints was missing for technical reasons.

#### Participant Characteristics

Multivariate Analysis of Variance (MANOVA) was used to check if participant characteristics and perceived fatigue after day 1 differed between age and practice groups. Since NASA-tlx ratings were not normally distributed even after log-transformation, separate Kruskal–Wallis tests were performed for younger and older adults to assess the effect of task difficulty on perceived mental workload. The effect of age on perceived mental workload was assessed by a separate Kruskal–Wallis test averaged over all difficulty levels. For all tests, a false discovery rate (FDR) correction was used to control for multiple comparisons.

#### Motor Performance and Neuroplasticity Assessments

Motor performance and TR-Power were assessed with multilevel linear random slope and intercept models with repeated measures (level 1) nested within subjects (level 2). First, effects of age and task difficulty on motor performance during the baseline phase were examined using a model that included the experimental variables Difficulty condition (LD, MD, HD), Practice group (P-LD, P-MD, P-HD), Age (young, old) and Block (Block 1, Block 2, Block 3) as fixed factors, as well as the Age^*^Block and Age^*^Difficulty condition interactions. Since motor performance is known to vary between individuals and especially between younger and older adults (Seidler et al., 2010), subject was entered as a random effect to allow for variation between subjects and thereby accounting for baseline differences. To assess changes in motor performance over time, multilevel linear random slope and intercept models were fitted with Age (young, old), Practice group (P-LD, P-MD, and P-HD), and Time (pre, post, and retention) as fixed factors and subject as a random effect. A full model was constructed, including the three-way interaction and all constituent terms. Because fast initial improvement took place during the baseline phase of the experiment and to fully capture the effects of task difficulty, pre-test motor performance was estimated from the first ten practice trials instead of the trials during the baseline test. Models were constructed separately for movement time and bandwidth error.

For TR-Power, separate models were constructed for the three ROIs (frontal, motor, parietal cortex) and two frequency bands (alpha, beta). TR-Power during the baseline phase was examined with a model including the fixed factors Difficulty condition (LD, MD, HD), Practice group (P-LD, P-MD, P-HD), Age (young, old), Block (Block 1, Block 2, Block 3), Hemisphere (contralateral, ipsilateral), Block^*^Age, Difficulty condition^*^Age, Hemisphere^*^Age, Difficulty condition^*^Hemisphere, and Difficulty condition^*^Hemisphere^*^Age, as well as a random effect for subject. Changes in TR-Power over time were assessed with a full model including the experimental variables Age (young, old), Practice group (P-LD, P-MD, P-HD), Time (baseline, post, retention) and Hemisphere (contralateral, ipsilateral), and all possible two-, three- and four-way interactions.

Models were fitted using restricted maximum likelihood. Normality of the distribution of the residuals was checked for all models with the Shapiro-Wilk test. In the case of a non-normal distribution, models were re-fitted with a log-transformed dependent variable. In addition, the influence of outliers on model outcomes was assessed using trimmed models excluding all data points with residuals ± 2 SDs from the mean, and robustness of effects was tested with a bootstrap validation (1,000 bootstrap samples, 95% confidence interval). Significance of fixed effect terms was assessed with Satterthwaite's method for degrees of freedom and F-statistic approximation (Luke, 2017). *Post-hoc* comparisons were tested using estimated marginal means from the models with a Tukey adjustment for multiple comparisons.

#### Relationship Between Motor Performance and EEG Data

Finally, we performed Pearson's correlation analysis to examine the relationship between motor performance and EEG data. To reduce the number of comparisons, correlation analyses followed a data-driven approach based on the results of the multilevel models. First, data from the baseline phase (i.e., all three difficulty conditions for all participants) were used to examine if there was a relationship between task difficulty and brain activity. Only EEG data for the brain cortices where a significant effect of task difficulty condition was found were correlated with behavior. In addition, if the effect of hemisphere in those cortices was not significant, TR-Power was first averaged across hemispheres. To examine age effects, correlations were calculated separately for younger and older adults. This resulted in four comparisons, which were corrected for multiple comparisons using an FDR correction. Second, the relationship between improvements in motor performance and change in TR-Power was investigated using change scores. Specifically, since the interaction effects with time, age, and practice group showed behavioral differences between the post and retention time-points (see results section), change scores for both behavioral and EEG data were calculated by subtracting post from retention values. Since TR-Power values represent the change in power during task execution relative to resting power (Gerloff et al., 1998) and lower alpha and beta power during task execution relative to a resting state is associated with higher brain activity (Pfurtscheller and Lopes da Silva, 1999), changes in TR-Power between two time-points are indicative of increased/decreased brain activity related to learning. Because we were specifically interested in the effect of task difficulty on learning, only the changes in TR-Power for the brain cortices where a significant Time^*^Practice group interaction was found were correlated with behavior. In addition, the change in TR-Power was averaged over both hemispheres if the Time^*^Hemisphere interaction was not significant. Again, an FDR correction was used to control for multiple comparisons (24 in total) and FDR-corrected *p*-values (p_cor_) are reported. The alpha level for all statistical tests was set to 0.05.

## Results

Table 1 shows the participants' characteristics, which were not different between age and practice groups except that older adults were somewhat shorter and physically more active than younger adults (main effect of age, *p* < 0.05).

**Table 1:** Participant characteristics.

	**Young**			**Old**		
	**P-LD**	**P-MD**	**P-HD**	**P-LD**	**P-MD**	**P-HD**
N	12	12	12	12	12	12
Sex (M/F)	6/6	5/7	5/7	7/5	7/5	6/6
Age (y)^a^	21.4 ± 1.5	20.9 ± 1.5	21.1 ± 1	70.3 ± 3.2	69.8 ± 3	71 ± 5.7
Height (cm)^a^	179.3 ± 10.3	178.7 ± 10.7	178.3 ± 11.6	173 ± 8.9	173.1 ± 9.9	175.2 ± 7.5
Weight (kg)	74.5 ± 11.2	74.4 ± 12.3	72.7 ± 15.6	84.3 ± 20.5	78 ± 12.7	70 ± 9
Laterality quotient^b^	89.5 ± 16	88 ± 13.6	91.9 ± 14.7	89.4 ± 17.1	87.9 ± 17.3	88.7 ± 22
Physical activity (MET-minutes/week)^a^^,^ ^c^	4580.7 ± 3182.5	3623.3 ± 1187.6	3405.4 ± 2591.3	5998.9 ± 4991.1	4872.5 ± 2581.5	5903.5 ± 3630.8
MMSE	N/A	N/A	N/A	29.3 ± 1	29.4 ± 0.9	28.8 ± 1.4
GARS	N/A	N/A	N/A	18.7 ± 2	18 ± 0	18.6 ± 1.7
Fatigue^d^	3.7 ± 2.2	3.5 ± 2.4	4.0 ± 2.3	2.8 ± 2.2	3.9 ± 2.5	3.8 ± 2.8

*Values are presented as mean ± standard deviations. P-LD, Practice with Low-Difficulty Task; P-MD, Practice with Medium-Difficulty Task; P-HD, Practice with High-Difficulty Task; MMSE, Mini-Mental State examination (>26 cognitively healthy); GARS, Groningen Activity Restriction Scale (18-72, higher scores means more restrictions with ADL activities); N/A, Not applicable*.

a *p < 0.05, young vs. old*.

b *Based on the Edinburgh Handedness Inventory [−100 (completely left-handed) to 100 (completely right-handed)]*.

c *Based on the short version of the International Physical Activity Questionnaire*.

d *Perceived fatigue after Day 1 measured on a Visual Analog Scale (0–10, higher scores means more fatigued)*.

### Perceived Fatigue and Mental Workload

A 3 (Difficulty condition) by 2 (Age) ANOVA showed that perceived fatigue at the end of day 1 did not differ between age or practice groups (Table 1). With regard to the NASA-tlx, Kruskal-Wallis tests confirmed that task difficulty affected the overall perceived mental workload only in younger adults (Supplementary Table 1 and Supplementary Figure 1) such that it was 49% higher in P-HD compared to P-LD (*p* = 0.006). Age did not affect the overall perceived mental workload (*p* = 0.26). The subscales of the NASA-tlx revealed differences between age groups (Supplementary Figure 1). Ratings from younger adults were 165% higher on the temporal demand subscale compared to older adults (*p* = 0.002), whereas ratings from older adults were 305% higher on the physical demand (*p* < 0.001) and 112% higher on the effort (*p* = 0.002) subscales compared to younger adults, suggesting that younger and older adults approached the motor task differently.

### Behavioral Data

**Figure 2:**
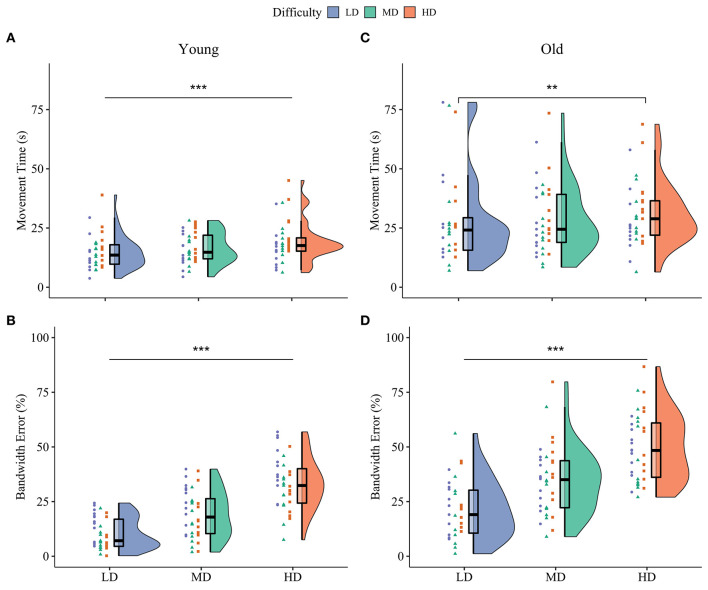
Baseline motor performance on the three difficulty conditions. Both movement time **(A)** and bandwidth error **(B)** differed between all three difficulty conditions in younger adults. For older adults, movement time only differed between the low and high difficulty condition **(C)**, while bandwidth error was different for all conditions **(D)**. Violin plots at the right side show the probability density, box-plots in the middle represents the median and interquartile range and dots on the left show individual raw data from the different practice groups. Blue: Low Difficulty (LD), green: Medium Difficulty (MD), orange: High Difficulty (HD). ^***^p < 0.001, ^**^p < 0.01.

**Figure 3:**
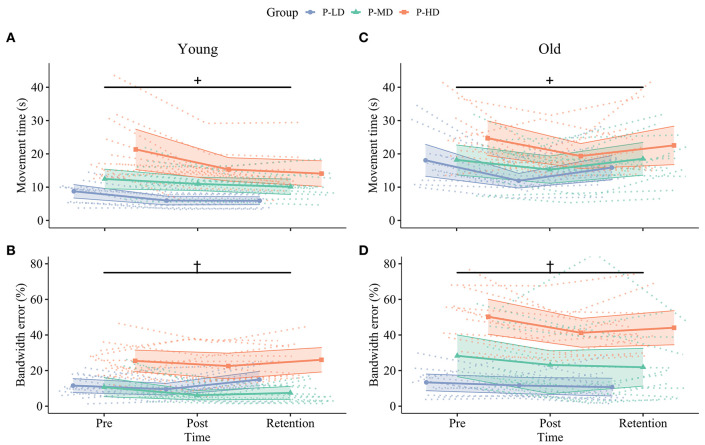
Change in speed (upper row, **A,C**) and accuracy (bottom row, **B,D**) over time for younger (left column, **A,B**) and older (right column, **C,D**) participants in the different practice groups. Dotted lines in the background show individual trajectories and shaded areas represent the 95% confidence intervals. Improvements in movement time from pre to post were greater in the P-LD and P-HD groups (+, significant Time^*^Practice group interaction, *p* = 0.01). Furthermore, younger adults only consolidated improvements in movement time **(A)**, while older adults only consolidated improvements in bandwidth error **(D)**. Practicing at a high difficulty level hindered motor skill retention of bandwidth error in older, but not younger adults (^†^, significant Time^*^Age^*^Practice group interaction, *p* = 0.03). Blue: Practice with Low Difficulty (P-LD), green: Practice with Medium Difficulty (P-MD), orange: Practice with High Difficulty (P-HD).

Table 2 and Figures 2, 3 summarize the motor performance data. The complete multilevel models are presented in Supplementary Table 2.

**Table 2:** F-tests of fixed effects for the behavioral data.

	**Movement time**			**Bandwidth error**		
	**F (df)**	**p**	**${\text{η}}_{\text{p}}^{2}$**	**F(df)**	**p**	**${\text{η}}_{\text{p}}^{\text{2}}$**
**Baseline**						
Block	177.7 (2,127.6)	<0.001^***^	0.74	50.9 (2,127.6)	<0.001^***^	0.44
Difficulty Condition	35.2 (2,127.6)	<0.001^***^	0.36	517.6 (2,127.9)	<0.001^***^	0.89
Practice Group	3.5 (2,68.1)	0.036^*^	0.09	1.5 (2,67.6)	0.23	0.04
Age	29.8 (1,68.2)	<0.001^***^	0.30	28.1 (1,67.6)	<0.001^***^	0.29
Age^*^Block	10.1 (2,127.6)	<0.001^***^	0.14	1.1 (2,127.9)	0.33	0.02
Age^*^Difficulty Condition	4.0 (2,127.6)	0.021^*^	0.06	4.1 (2,127.9)	0.018^*^	0.06
**Learning**						
Time	46.2 (2,132)	<0.001^***^	0.41	17.2 (2,127.1)	<0.001^***^	0.21
Age	37.0 (1,66)	<0.001^***^	0.36	14.5 (1,65.6)	<0.001^***^	0.18
Practice Group	19.7 (2,66)	<0.001^***^	0.37	27.0 (2,65.6)	<0.001^***^	0.45
Time^*^Age	13.4 (2,132)	<0.001^***^	0.17	3.3 (2,127.1)	0.04^*^	0.05
Time^*^Practice Group	3.4 (4,132)	0.012^*^	0.09	3.6 (4,127.1)	0.008^**^	0.10
Age^*^Practice Group	3.4 (2,66)	0.04^*^	0.09	4.6 (2,65.6)	0.01^*^	0.12
Time^*^Age^*^Practice Group	0.4 (4,132)	0.84	0.01	2.8 (4,127.1)	0.027^*^	0.08

* *p < 0.05*;

** *p < 0.01*;

*** *p < 0.001*.

#### Baseline

##### Movement Time

The multilevel model of motor performance showed that older adults moved 73% slower than younger adults during the baseline phase (Age main effect, *p* < 0.001, ${\text{η}}_{\text{p}}^{2}$ = 0.30). A significant main effect of Block (*p* < 0.001, ${\text{η}}_{\text{p}}^{2}$ = 0.74) indicated fast initial improvement over the baseline trials. *Post hoc* comparisons of the Age^*^Difficulty Condition interaction showed that in younger adults, movement time increased between all three difficulty conditions, indicating that younger adults moved slower as task difficulty increased (LD/MD: 11% increase, *p* = 0.004; LD/HD: 30% increase, *p* < 0.001; MD/HD: 17% increase, *p* < 0.001). In older adults, movement time only increased in the high difficulty condition (LD/MD: 2% increase, *p* = 0.53; LD/HD: 11% increase, *p* < 0.001; MD/HD: 9% increase, *p* = 0.016; Figures 2A,C). There was a main effect of Practice group for movement time (*p* = 0.04, ${\text{η}}_{\text{p}}^{2}$ = 0.09), suggesting that participants in P-HD moved slower compared to P-LD and P-MD. However, none of the *post-hoc* tests reached significance (P-LD/P-HD: *p* = 0.07; P-MD/P-HD: *p* = 0.06). An Age^*^Block interaction for movement time revealed that the improvement from block 1 to block 2 was higher in older (35%) compared to younger (20%) adults (*p* < 0.001, ${\text{η}}_{\text{p}}^{2}$ = 0.14).

##### Bandwidth Error

Overall, bandwidth error was 71% higher in older than younger adults during the baseline phase (Age main effect, *p* < 0.001, ${\text{η}}_{\text{p}}^{2}$ = 0.29). Bandwidth error differed between all three difficulty conditions in both age groups, indicating the successful manipulation of task difficulty (Difficulty Condition main effect, *p* < 0.001, ${\text{η}}_{\text{p}}^{2}$ = 0.89; Figures 2B,D). *Post-hoc* tests showed that bandwidth error increased more between the low difficulty condition and the other two difficulty conditions in older compared to younger adults (LD/MD: *p* = 0.03, LD/HD: *p* = 0.007). Similar to movement time, a significant main effect of Block indicated fast initial improvement over the baseline trials (*p* < 0.001, ${\text{η}}_{\text{p}}^{2}$ = 0.44).

#### Learning

##### Movement Time

The multilevel model assessing learning revealed Time^*^Age (${\text{η}}_{\text{p}}^{2}$ = 0.17) and Time^*^Practice group (${\text{η}}_{\text{p}}^{2}$ = 0.09) interactions for movement time. While movement time improved in both age groups at similar rates from pre to post across difficulty levels (both 24% improvement, *p* < 0.001), only the younger adults maintained this improvement at 24 h retention (Time^*^Age interaction; 7% change post to retention, *p* = 0.25; Figure 3A), while the movement time from older adults reverted to baseline levels (−22% change post to retention, *p* < 0.001; Figure 3C). In addition, the Time^*^Practice group interaction (${\text{η}}_{\text{p}}^{2}$ = 0.09) showed that across age groups, learning rates from pre to post were higher in P-LD (33% improvement, *p* < 0.001) and P-HD (25% improvement, *p* < 0.001) compared to P-MD (14% improvement, *p* = 0.01).

##### Bandwidth Error

A significant Time^*^Age^*^Practice group interaction indicated that task difficulty affected improvements in bandwidth error differently in younger and older adults (*p* = 0.03, ${\text{η}}_{\text{p}}^{2}$ = 0.08). For younger adults, only the group practicing at the medium difficulty level improved bandwidth error (43% improvement pre-post, *p* = 0.002; Figure 3B). In contrast, younger adults in the P-LD and P-HD groups did not improve bandwidth error significantly over practice (P-LD: 18% improvement pre-post, *p* = 0.50; P-HD: 11% improvement pre-post, *p* = 0.23), and performance from P-LD at retention was even 30% worse compared to the pre-test (*p* = 0.047). For older adults, improvements from pre to post ranged from 13 to 33% between practice groups. Older P-LD and P-MD were able to maintain this improvement at retention (P-LD: 20% difference pre-retention, *p* = 0.012; P-MD: 23% difference pre-retention, *p* = 0.008), while performance from P-HD deteriorated with 7% from post to retention (12% difference pre-retention, *p* = 0.54; Figure 3D).

In summary, both age and task difficulty affected motor skill acquisition and retention. Young participants who practiced at the low and high difficulty levels improved tracing speed, but not accuracy from pre to post. In contrast, young participants who practiced at the medium difficulty level improved tracing accuracy but showed the least improvement in speed. Older participants improved both speed and accuracy across all difficulty levels. At retention, younger adults only consolidated improvements in speed, while older adults only consolidated improvements in accuracy. Motor skill retention was dependent on task difficulty only in older adults. Older adults that practiced at the low or medium, but not the high, difficulty levels were able to maintain improvements in accuracy at retention.

### EEG Power Analyses

**Figure 4:**
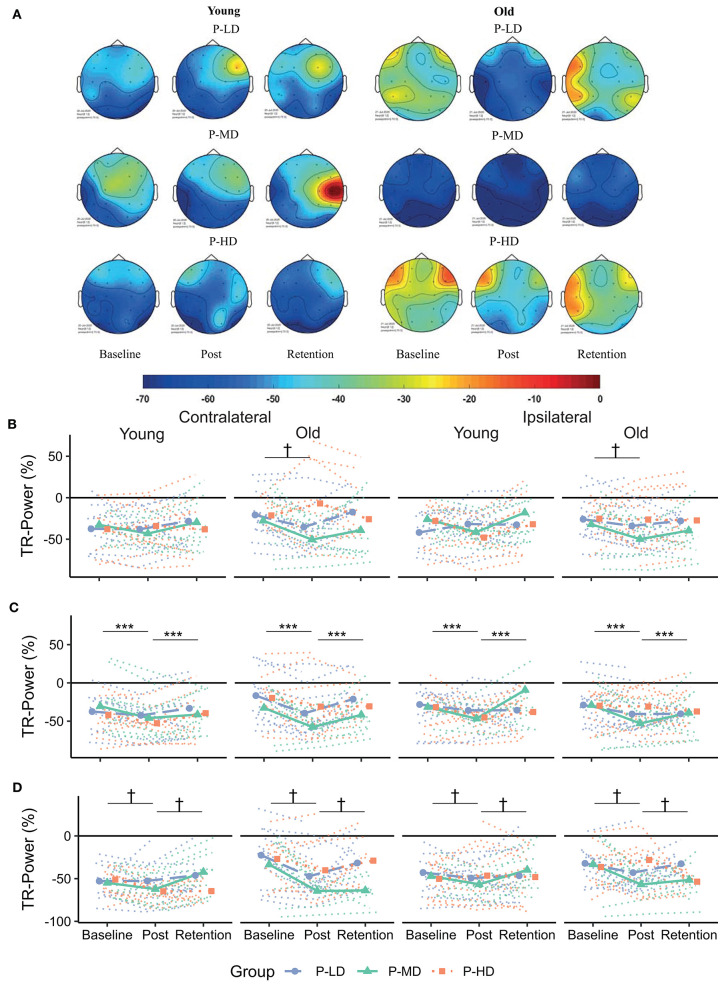
**(A)** Topographical plots showing the distribution of alpha TR-Power over the ROIs for the different age and practice groups. Cooler colors represent lower TR-Power (i.e., higher activity). Black dots represent the electrodes used to define the ROIs. **(B–D)** Change in TR-Power over time for the frontal **(B)**, motor **(C)** and parietal **(D)** cortex. Dotted lines represent individual trajectories. Overall, alpha power was more lateralized (i.e., more negative) contralateral to the moving hand for younger compared to older adults. Over the frontal and parietal cortices, TR-Power decreased from baseline to the post-test. This decrease was greatest for older adults practicing at a low or medium difficulty level. P-LD, Practice with Low Difficulty (blue dots); P-MD, Practice with Medium Difficulty (green triangles); P-HD, Practice with High Difficulty (orange squares). ^***^Main effect of time at *p* < 0.001; ^†^Time^*^Age^*^Practice group interaction at *p* < 0.05.

**Figure 5:**
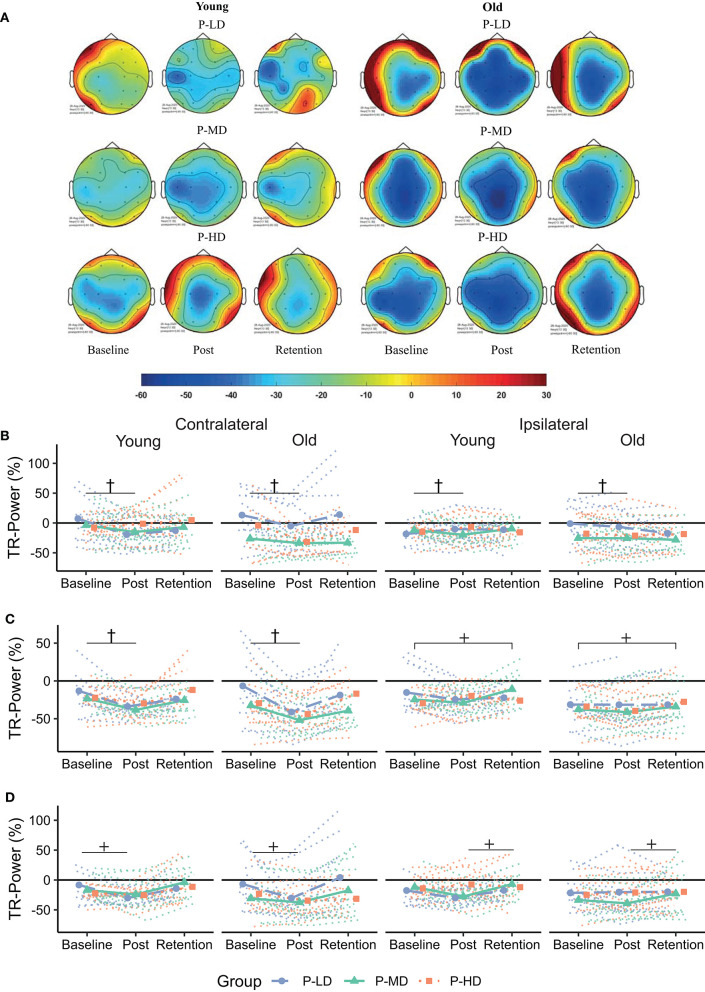
**(A)** Topographical plots showing the distribution of beta TR-Power over the ROIs for the different age and practice groups. Cooler colors represent lower TR-Power (i.e., higher activity). Black dots represent the electrodes used to define the ROIs. **(B–D)** Change in TR-Power over time for the frontal **(B)**, motor **(C)**, and parietal **(D)** cortex. Dotted lines represent individual trajectories. Overall, there was a decrease in beta TR-Power from baseline to the post-test. This decrease was specific for participants who practiced at the low or medium difficulty levels over the frontal and motor cortices and lateralized to the contralateral hemisphere over the motor and parietal cortices. P-LD, Practice with Low Difficulty (blue dots); P-MD, Practice with Medium Difficulty (green triangles), P-HD, Practice with High Difficulty (orange squares). ^†^Time^*^Practice group interaction at *p* < 0.05; ^+^Time^*^Hemsphere interaction at *p* < 0.05.

Table 3 and Figures 4, 5 summarize the TR-Power data. Supplementary Table 3 shows the complete multilevel models.

**Table 3:** F-tests of fixed effects for the TR-Power data.

**Baseline**									
**Alpha**	**Frontal**			**Motor**			**Parietal**		
	**F (df)**	**p**	${\text{η}}_{\text{p}}^{\text{2}}$	**F (df)**	**p**	${\text{η}}_{\text{p}}^{\text{2}}$	**F (df)**	**p**	${\text{η}}_{\text{p}}^{\text{2}}$
Block	2.8 (2,321.3)	0.06	0.03	2.5 (2,321.2)	0.08	0.02	2.7 (2,315.2)	0.07	0.02
Difficulty condition	0.5 (2,321.3)	0.6	0.003	2.5 (2,321.2)	0.08	0.02	0.08 (2,315.2)	0.9	<0.001
Hemisphere	3.6 (1,321.2)	0.06	0.01	5.6 (1,321.2)	0.02^*^	0.02	3.8 (1,315.1)	0.05	0.01
Practice Group	0.08 (2,68.1)	0.9	0.002	0.1 (2,68)	0.9	0.003	0.3 (2,67)	0.8	0.008
Age	1.8 (1,68.1)	0.2	0.03	2.6 (1,68)	0.1	0.04	4.6 (1,67)	0.03^*^	0.06
Age^*^Block	2.9 (2,321.3)	0.06	0.02	0.9 (2,321.2)	0.4	0.006	0.8 (2,315.2)	0.5	0.005
Age^*^Difficulty Condition	0.6 (2,321.3)	0.6	0.003	0.9 (2,321.2)	0.4	0.006	2.4 (2,315.2)	0.09	0.02
Age^*^Hemisphere	8.7 (1,321.2)	0.003^**^	0.03	19.4 (1,321.2)	<0.001^***^	0.06	4.1 (1,315.1)	0.04^*^	0.01
Difficulty Condition ^*^Hemisphere	0.1 (2,321.2)	0.9	<0.001	0.5 (2,321.1)	0.6	0.003	1.5 (2,315.2)	0.2	0.009
Age^*^Difficulty Condition^*^Hemisphere	0.1 (2,321.2)	0.9	<0.001	2.1 (2,321.1)	0.1	0.01	1.8 (2,315.2)	0.2	0.01
**Beta**	**Frontal**			**Motor**			**Parietal**		
Block	1.5 (2,322.9)	0.2	0.009	1.4 (2,319.1)	0.2	0.009	1.0 (2,324.9)	0.4	0.006
Difficulty Condition	3.8 (2,322.9)	0.02^*^	0.02	0.6 (2,319.2)	0.5	0.004	0.5 (2,324.9)	0.6	0.003
Hemisphere	0.03 (1,322.5)	0.9	<0.001	0.7 (1,319)	0.4	0.002	0.6 (1,324.9)	0.4	0.002
Practice Group	1.6 (2,67.4)	0.2	0.05	1.2 (2,66.5)	0.3	0.03	0.4 (2,66.8)	0.7	0.01
Age	0.02 (1,67.4)	0.9	<0.001	0.5 (1,66.5)	0.5	0.008	0.8 (1,66.8)	0.4	0.01
Age^*^Block	2.3 (2,322.9)	0.1	0.01	0.5 (2,319.1)	0.6	0.003	0.1 (2,324.9)	0.9	<0.001
Age^*^Difficulty Condition	0.3 (2,322.9)	0.7	0.002	0.04 (2,319.2)	1	<0.001	1.6 (2,324.9)	0.2	0.009
Age^*^Hemisphere	0.5 (1,322.5)	0.5	0.001	0.8 (1,319)	0.4	0.002	1.4 (1,324.9)	0.2	0.004
Difficulty Condition ^*^Hemisphere	2.2 (2,322.6)	0.1	0.01	0.04 (2,319.1)	1	<0.001	0.2 (2,324.9)	0.8	0.001
Age^*^Difficulty Condition^*^Hemisphere	0.2 (2,322.7)	0.9	<0.001	0.4 (2,319.1)	0.7	0.003	0.08 (2,324.9)	0.9	<0.001
**Learning**									
**Alpha**	**Frontal**			**Motor**			**Parietal**		
	**F (df)**	**p**	${\text{η}}_{\text{p}}^{2}$	**F (df)**	**p**	${\text{η}}_{\text{p}}^{2}$	**F (df)**	**p**	${\text{η}}_{\text{p}}^{2}$
Time	7.8 (2,300.8)	<0.001^***^	0.05	19.0 (2,302.1)	<0.001^***^	0.11	24.3 (2,296.0)	<0.001^***^	0.14
Hemisphere	0.04 (1,300.1)	0.8	<0.001	1.4 (1,301.7)	0.2	0.005	0.8 (1,296)	0.4	0.003
Age	0.003 (1,65.8)	1	<0.001	0.09 (1,65.1)	0.8	0.001	1.8 (1,65.2)	0.2	0.03
Practice Group	0.4 (2,65.8)	0.7	0.01	0.3 (2,65.1)	0.7	0.009	0.5 (2,65.2)	0.6	0.02
Time^*^Hemisphere	0.7 (2,300.1)	0.5	0.005	1.5 (2,301.5)	0.2	0.01	3.8 (2,295.7)	0.02^*^	0.03
Time^*^Age	3.2 (2,300.8)	0.04^*^	0.02	2.7 (2,302.1)	0.07	0.02	2.3 (2,296)	0.1	0.02
Time^*^ Practice Group	3.1 (4,300.8)	0.02^*^	0.04	1.3 (4,302.1)	0.3	0.02	3.5 (4,296)	0.008^**^	0.04
Age^*^Hemisphere	5.6 (1,300.1)	0.02^*^	0.02	14.6 (1,301.7)	<0.001^***^	0.05	2.7 (1,296)	0.1	0.009
Practice Group^*^Hemisphere	0.9 (2,300.1)	0.4	0.006	4.5 (2,301.7)	0.01^*^	0.03	2.2 (2,296)	0.1	0.01
Age^*^ Practice Group	0.6 (2,65.8)	0.5	0.02	0.8 (2,65.1)	0.4	0.02	0.9 (2,65.2)	0.4	0.03
Time^*^Hemisphere^*^Age	0.2 (2,300.1)	0.8	0.001	1.4 (2,301.5)	0.2	0.009	1.2 (2,295.7)	0.3	0.008
Time^*^Hemisphere^*^Practice Group	0.8 (4,300.1)	0.6	0.01	0.8 (4,301.5)	0.5	0.01	1.9 (4,295.7)	0.1	0.03
Time^*^Age^*^Practice Group	2.9 (4,300.8)	0.02^*^	0.04	2.4 (4,302.1)	0.05	0.03	6.6 (4,296)	<0.001^***^	0.08
Hemisphere^*^Age^*^Practice Group	0.3 (2,300.1)	0.8	0.002	0.8 (2,301.7)	0.5	0.005	2.0 (2,296)	0.1	0.01
Time^*^Hemisphere^*^Age^*^ Practice Group	0.5 (4,300.1)	0.8	0.006	0.8 (4,301.5)	0.5	0.01	0.7 (4,296)	0.6	0.01
**Beta**	**Frontal**			**Motor**			**Parietal**		
Time	11.0 (2,294.8)	<0.001^***^	0.07	31.8 (2,294.9)	<0.001^***^	0.18	30.5 (2,294.8)	<0.001^***^	0.17
Hemisphere	0.1 (1,294.3)	0.7	<0.001	0.07 (1,294.2)	0.8	<0.001	0.8 (1,294.6)	0.4	0.003
Age	0.3 (1,65.8)	0.6	0.005	3.3 (1,65.8)	0.07	0.05	3.7 (1,65.9)	0.06	0.05
Practice Group	1.1 (2,65.8)	0.3	0.03	0.7 (2,65.8)	0.5	0.02	0.8 (2,65.9)	0.5	0.02
Time^*^Hemisphere	0.9 (2,294.3)	0.4	0.006	3.5 (2,294.2)	0.03^*^	0.02	3.6 (2,294.6)	0.03^*^	0.02
Time^*^Age	0.6 (2,294.8)	0.5	0.004	2.0 (2,294.9)	0.1	0.01	1.7 (2,294.8)	0.2	0.01
Time^*^ Practice Group	4.1 (4,294.8)	0.003^**^	0.05	2.6 (4,294.8)	0.04^*^	0.03	0.9 (4,294.8)	0.5	0.01
Age^*^Hemisphere	0.3 (1,294.3)	0.6	0.001	5.4 (1,294.2)	0.02^*^	0.02	0.05 (1,294.6)	0.8	<0.001
Practice Group^*^Hemisphere	0.8 (2,294.3)	0.4	0.006	1.4 (2,294.2)	0.3	0.009	0.05 (2,294.6)	0.9	<0.001
Age^*^ Practice Group	0.7 (2,65.8)	0.5	0.02	0.4 (2,65.8)	0.7	0.01	0.4 (2,65.9)	0.7	0.01
Time^*^Hemisphere^*^Age	0.4 (2,294.3)	0.7	0.003	1.2 (2,294.2)	0.3	0.008	1.6 (2,294.6)	0.2	0.01
Time^*^Hemisphere^*^Practice Group	2.4 (4,294.3)	0.05	0.03	1.5 (4,294.2)	0.2	0.02	1.6 (4,294.6)	0.2	0.02
Time^*^Age^*^Practice Group	0.4 (4,294.8)	0.8	0.006	1.3 (4,294.8)	0.3	0.02	1.0 (4,294.8)	0.4	0.01
Hemisphere^*^Age^*^Practice Group	0.2 (2,294.3)	0.8	0.001	1.6 (2,294.2)	0.2	0.01	1.9 (2,294.6)	0.2	0.01
Time^*^Hemisphere^*^Age^*^Practice Group	0.5 (4,294.3)	0.7	0.007	0.3 (4,294.2)	0.8	0.005	0.9 (4,294.6)	0.4	0.01

* *p < 0.05*;

** *p < 0.01*;

*** *p < 0.001*.

#### Baseline

##### Alpha Band

During the baseline phase, *post-hoc* comparisons showed that alpha TR-Power over the frontal and motor cortices was respectively, 14 and 15% higher (i.e., less activity) in the ipsi- compared to the contralateral hemisphere in younger adults (both *p* < 0.001), while alpha TR-Power did not differ between hemispheres in older adults (Hemisphere^*^Age interaction, ${\text{η}}_{\text{p}}^{2}$ = 0.06; Frontal: *p* = 0.5; Motor: *p* = 0.4). An opposite effect was seen over the parietal cortex, where alpha TR-Power was 11% lower in the ipsi- compared to the contralateral hemisphere in older adults (*p* < 0.001), but did not differ between hemispheres in younger adults (*p* = 0.9). No significant differences in alpha TR-Power were found between difficulty conditions, practice groups, or baseline blocks (Table 3).

##### Beta Band

Across age groups, practice groups, and hemispheres, beta TR-Power over the frontal cortex was lower during the high (mean ± SD: −3.25 ± 41.7%), compared to the low (1.67 ± 46.0%) or medium (3.48 ± 55.5%) difficulty conditions (Difficulty Condition main effect: *p* = 0.02, ${\text{η}}_{\text{p}}^{2}$ = 0.02). All other main and interaction terms in the model were not significant, indicating no significant differences in beta TR-Power between practice or age groups, baseline blocks, or hemispheres.

#### Learning

##### Alpha Band

Effects of age and task difficulty on changes in alpha TR-Power over the practice period differed by ROI (Figure 4), as revealed by the multilevel model for learning. Over the frontal cortex, TR-Power decreased on average 48 and 58% across hemispheres directly after practice for old P-LD and P-MD (*p* = 0.03 and *p* < 0.001, respectively), but did not change for old P-HD (*p* = 0.9) or any of the young practice groups (Time^*^Age^*^Practice group interaction: *p* = 0.02, ${\text{η}}_{\text{p}}^{2}$ = 0.04). At 24 h retention, frontal alpha TR-Power did not significantly differ from baseline levels for any of the groups (all *p* > 0.05). No effects of age or task difficulty were seen over the motor cortex, where alpha TR-Power decreased from baseline to post (*p* < 0.001) and increased again from day 1 to day 2 (*p* < 0.001) across hemispheres in all groups (Time main effect: *p* < 0.001, ${\text{η}}_{\text{p}}^{2}$ = 0.11). Over the parietal cortex, both age and task difficulty affected changes in TR-Power over time (Time^*^Age^*^Practice group interaction: *p* < 0.001, ${\text{η}}_{\text{p}}^{2}$ = 0.08). For younger adults, changes in alpha TR-Power over time were only seen in P-MD. For this group, TR-Power decreased 35% across hemispheres directly after learning (*p* = 0.002) and increased back to baseline levels from day 1 to day 2 (11% difference baseline-retention, *p* = 0.53). For older adults, TR-Power across hemispheres decreased 84.7% directly after learning for P-LD and 61% for P-MD (both *p* < 0.001), but did not change for P-HD (*p* = 0.82). At 24 h retention, TR-Power increased back to baseline levels for P-LD (-19% difference baseline-retention, *p* = 0.9), while it remained low for P-MD (−55% difference baseline-retention, *p* < 0.001).

In summary, effects of age and task difficulty on changes in alpha TR-Power over time were seen over the frontal and parietal cortices, where a decrease in TR-Power directly after learning was greatest for older adults practicing the task at a low or medium difficulty level.

##### Beta Band

Overall, the multilevel model for learning showed that beta TR-Power decreased directly after practice and increased back to baseline levels at 24 h retention (Figure 5). Over the frontal and motor cortices, this practice-related decrease was dependent on task difficulty, but not age. Time^*^Practice Group interactions in these cortices (Frontal: *p* = 0.003, ${\text{η}}_{\text{p}}^{2}$ = 0.05; Motor: *p* = 0.04, ${\text{η}}_{\text{p}}^{2}$ = 0.03) showed a decrease in TR-Power across hemispheres from baseline to post in P-LD (Frontal: 142% decrease, *p* < 0.001; Motor: 81% decrease, *p* < 0.001) and P-MD (Frontal: 195% decrease, *p* = 0.003; Motor: 41% decrease, *p* < 0.001), but not in P-HD (Frontal: 101% decrease, *p* = 0.89; Motor: 43% decrease, *p* = 0.32). Furthermore, a Time^*^Hemisphere interaction in the motor and parietal cortices (both *p* = 0.03, ${\text{η}}_{\text{p}}^{2}$ = 0.02) indicated that the practice-related decrease from baseline to post was higher in the contra- compared to the ipsilateral hemisphere (Motor: *p* = 0.01; Parietal: *p* = 0.01). In contrast, at day 2 beta TR-Power in the ipsilateral motor cortex was 24% higher than at baseline (*p* = 0.02), while beta TR-Power in the contralateral motor cortex at retention did not differ from baseline (*p* = 1.0).

Taken together, there was a decrease in beta TR-Power directly after practice, which was specific for P-LD and P-MD in the frontal and motor cortices and lateralized to the contralateral hemisphere in the motor and parietal cortices. At 24 h retention, TR-Power was higher compared to baseline in the ipsilateral motor cortex for both age groups.

### Correlation Analyses

First, correlation analyses were performed with data from the baseline phase to examine if there was a relationship between task difficulty and brain activity. During the baseline phase, an effect of difficulty condition, but not hemisphere, was seen for the frontal beta TR-Power (see section Baseline). Therefore, we correlated average frontal beta TR-Power across hemispheres with behavior. After multiple comparisons correction, no significant correlations were found.

Second, the relationship between improvements in motor performance and change in TR-Power was investigated. Since the interaction effects with time, age, and practice group showed behavioral differences between the post and retention time-points (see section Behavioral Data, Learning), only the change scores between post and retention were used for the correlation analysis. A Time^*^Practice group interaction was found in four of the six ROIs (frontal alpha, parietal alpha, frontal beta, and motor beta; see section EEG Power Analyses, Learning), two of which also showed a significant Time^*^Hemisphere interaction (parietal alpha and motor beta). For the remaining two ROIs, data were averaged over the two hemispheres before they were correlated to behavior. None of the correlations reached significance after FDR correction.

## Discussion

The present study aimed to determine the effects of age and task difficulty on motor learning and associated neural plasticity. In line with the hypothesis, both age and task difficulty affected motor skill acquisition and retention. Firstly, we found an age-related deterioration in motor performance at baseline that was more pronounced with increasing task difficulty. Secondly, task difficulty differentially affected motor skill acquisition and retention in older compared with younger adults: while motor skill acquisition was only affected by task difficulty in younger, but not older adults, task difficulty affected motor skill retention in older, but not younger adults. Thirdly, at retention, younger adults only consolidated improvements in speed, while older adults only consolidated improvements in accuracy. Fourthly, we found a more bilateral alpha activity pattern for older compared to younger adults during motor performance independent of task difficulty. Lastly, TR-Power decreased from baseline to post and reverted to baseline levels at 24-h retention. The magnitude of this decrease was dependent on age, task difficulty, and the region of interest.

### Age-Related Deterioration in Motor Performance Increases With Task Difficulty

As expected, older compared with younger adults executed the star-tracing skill more slowly and less accurately. In addition, the age-related deterioration in movement accuracy was most pronounced in the high difficulty condition (Figure 2D). This is in line with previous studies showing an increased age-related decline in motor performance with increasing task difficulty (Smith et al., 1999; Bangert et al., 2010). Together, these studies provide experimental support for the suggestion that the CRUNCH model is not only applicable in the cognitive but also in the motor domain.

### Age and Task Difficulty Affect Motor Skill Acquisition and Retention

Skill acquisition did not differ between younger and older adults, corroborating findings from earlier studies (Cirillo et al., 2011; Berghuis et al., 2019). Interestingly, task difficulty did only impact motor skill acquisition in younger, but not in older adults. Young participants who practiced at the low and high difficulty levels improved tracing speed, but not accuracy from pre to post. In contrast, young participants who practiced at the medium difficulty level improved tracing accuracy but showed the least improvement in speed (Figures 3A,B). Earlier research showed that individuals differ in the strategy they use to achieve the task goal in a redundant motor task, where motor performance can be improved through multiple solutions (i.e., spatial and temporal) (King et al., 2012). The current results extend this finding by suggesting that the strategy in younger adults is not only dependent on individual factors, but also on external factors such as task difficulty. However, since each difficulty group consisted of different individuals, further research with task difficulty as a within-subjects factor is needed to confirm this hypothesis. Furthermore, the observation that motor performance improved irrespective of practice group suggests that theoretical models stating that heightened motor and cognitive demands act as a stimulus for learning when task difficulty increases (Locke and Latham, 2002; Guadagnoli and Lee, 2004) are not sufficient to explain the effect of task difficulty on skill acquisition of a redundant motor task.

Not only task difficulty but also age affected the performance and learning of the mirror star tracing task. While younger adults only consolidated (i.e., stabilization of performance at retention relative to post) improvements in performance speed, consolidation in older adults was limited to performance accuracy (Figures 3A,D). In addition, older vs. younger adults had lower ratings on the temporal demand subscale of the NASA-tlx. Prioritization of accuracy over speed in older adults has been reported previously and is suggested to reflect a learned strategy to maintain precision of movements and be able to process online feedback (Lamb et al., 2016). Thus, it seems that while learning a mirror star tracing task, younger adults focus more on improving the temporal aspect of the movement, whereas older adults focus more on improving the spatial aspects.

In agreement with the hypothesis, task difficulty affected motor skill retention specifically in older adults. Older adults that practiced at the low or medium, but not the high, difficulty levels were able to maintain improvements in accuracy at retention (Figure 3D). Together with the increased age-related deterioration of motor performance at the high difficulty level, these results support predictions from the CRUNCH model as well as from the optimal challenge point framework, which state that when a task is too difficult, ceiling levels in terms of neural resources are reached and this might hamper learning (Guadagnoli and Lee, 2004; Reuter-Lorenz and Cappell, 2008). This finding also agrees with an earlier study using a force tracking task (Onushko et al., 2014) and confirms that impaired skill retention of tasks with a high level of difficulty is an age-specific phenomenon.

Since sleep is known to affect the overnight retention of motor skills (Robertson et al., 2004; Schmid et al., 2020), altered sleep architecture with increasing age possibly contributes to the impaired skill retention of the high difficulty task in older adults (Ohayon et al., 2004). There is growing consensus that sleep spindles play an important role in overnight memory consolidation (Fogel and Smith, 2011; van Schalkwijk et al., 2020) and it is known that the duration, amplitude, and density of sleep spindles is reduced with increasing age (Martin et al., 2013; Peters et al., 2014). Indeed, the relationship between sleep spindles and motor memory consolidation is different for younger and older adults (Fogel et al., 2014). Furthermore, the difficulty of a declarative memory task affected sleep spindle density in a post-training daytime nap (Schmidt et al., 2006). One might therefore speculate that the density and duration of sleep spindles are also affected by the difficulty of a motor task. Further studies are needed to examine whether motor task difficulty alters the relationship between age, sleep architecture, and motor memory consolidation.

### More Bilateral Activity in Older vs. Younger Adults

Consistent with previous data, activity patterns during motor performance were different in older compared to younger adults (Berghuis et al., 2019; Larivière et al., 2019). Specifically, over the frontal and motor cortices, a more bilateral activity pattern was seen for alpha power during task execution for older compared to younger adults. Moreover, alpha activity over the parietal cortex was higher over the contra- compared to the ipsilateral hemisphere in older but not younger adults. Together, these results show a more bilateral and anterior pattern of alpha activity in older compared to younger adults, in line with the HAROLD and PASA models (Cabeza, 2002; Davis et al., 2008).

Bilateral frontal beta TR-Power was lower during the high, compared to the low or medium difficulty conditions in both age groups. Bilateral frontal recruitment has been associated with cognitive control, and an increase in bilateral frontal activity might therefore indicate increased cognitive demand associated with higher task difficulty, in accordance with earlier studies (Manganotti et al., 1998; Verstynen et al., 2005).

### Practice-Related Changes in Task-Related Power Are Dependent on Age and Task Difficulty

In general, alpha and beta TR-Power decreased from baseline to post. A decrease of TR-Power after a period of motor practice has been reported earlier and is interpreted as a marker of early processes of neural plasticity (Boonstra et al., 2007; Nakano et al., 2013). Interestingly, while previous studies have reported reduced neural plasticity in older compared to younger adults (Mary et al., 2015; Rueda-Delgado et al., 2019), we found greater changes in alpha TR-Power in older compared to younger adults (Figure 4). A decrease of alpha power during execution of motor tasks relative to resting conditions (i.e., lower TR-Power) has been related to cognitive-motor processing, attention, and effort (Klimesch, 1999; van Wijk et al., 2012). As such, it is possible that the decrease in TR-Power in older adults reflects a compensatory mechanism necessary to cope with the task demand and improve motor performance. The data allude to the possibility that healthy older adults in the present study had a reserve but perhaps unused plasticity in the alpha band, which they were able to exploit, when called for, during motor practice.

In line with the view that heightened activity in older adults reflects a compensatory mechanism, the decrease in alpha TR-Power in older adults was dependent on task difficulty, so that it was only present in P-LD and P-MD, but not in P-HD. Together with the impaired retention, this finding further supports the idea that practicing a motor task at a high difficulty level is not beneficial for motor learning in older adults (Bootsma et al., 2020). Since decreased TR-Power after motor practice can be seen as a marker of early neural plasticity processes, the absence of change for the old P-HD group might indicate a failure to engage neural plasticity, leading to impaired retention of the learned skill.

An alternative explanation for the age-dependent change of alpha TR-Power might be that a decrease of alpha TR-Power is specifically related to an increase in the accuracy of motor performance. While younger adults became faster after practice, older adults became more accurate. Furthermore, young P-MD was the only young group in which parietal alpha TR-Power decreased from baseline to post, while it was also the only young group that improved accuracy over the practice period. As noted before, modulation of alpha power during task relative to resting conditions has been related to attentional processes (van Wijk et al., 2012). In a go/no go task, a greater allocation of visual-spatial attention was found in the group that prioritized accuracy over speed (Perri et al., 2014), providing a possible explanation for the potential relationship between decreased alpha TR-Power and increased accuracy. It should however be noted that we did not find significant correlations between improvements in accuracy and changes in alpha TR-Power and therefore, this hypothesis is merely speculative at this stage and warrants further investigation.

At 24-h retention, beta TR-Power over the ipsilateral motor cortex was higher compared to baseline across all age and practice groups, indicating a shift to a more lateralized activity pattern (Figure 5). A similar finding was found in a previous study using a visuomotor tracking task, where smaller pre-training beta activity over the ipsilateral cortex was related to better performance after practice (Espenhahn et al., 2019). Although the functional role of the ipsilateral cortex in motor control and learning is still under debate (Uehara and Funase, 2014; Barany et al., 2020; Cabibel et al., 2020), these results are consistent with the view that a more lateralized activity pattern is beneficial for motor performance (Takeuchi et al., 2012).

Together, the power data show reduced neural plasticity in older adults specifically after practice at the high difficulty level, while no age-related deterioration in neural plasticity was present after practice at low or medium difficulty levels. Although the changes in task-related power are in line with the changes in motor performance, we did not find any significant correlations between behavioral and neural measures. It is possible that none of the correlations survived the multiple comparison correction due to the relatively small sample size and high between-subjects variability. In line with the view that increased brain activity after motor practice is a compensatory mechanism in older adults, we did observe a trend for a correlation suggesting that older adults who showed the least increase in TR-Power also showed the least deterioration in motor performance from post to retention (data not shown). However, more research with larger samples is needed to verify possible relations between changes in TR-Power and changes in motor performance.

### Clinical Applications and Future Directions

Understanding the effects of age on motor learning and practice-related plasticity can aid to develop rehabilitation practice schemes for patients recovering from movement impairments (Ward, 2017). Specifically, the observation that older adults only consolidated improvements in motor performance after practice with low or medium difficulty levels suggests that older adults in rehabilitation settings would benefit more when practicing under conditions with lower difficulty levels. Furthermore, our data imply that age and task difficulty might affect spatial and temporal aspects of a motor skill differently, emphasizing the need to tailor motor task characteristics to the desired rehabilitation outcomes. Task difficulty could potentially be added to the variables used to dose motor learning in clinical rehabilitation (Wang et al., 2020). To apply the current results to clinical settings, further studies need to assess if the improvements after practice with low or medium difficulty levels are also consolidated over longer retention periods. Promising results have been found by several studies showing at least partial retention up to 5 years after motor practice (Rodrigue et al., 2005; Schaefer and Duff, 2015). If and how this long-term retention applies to the current results, as well as how the current results apply to other motor skills, activities of daily living, and clinical populations such as stroke patients, are important targets for future research.

### Limitations

Previous studies have suggested that age-related deteriorations of motor performance and practice-related plasticity follow a more gradual decline throughout the age spectrum (Pascual-Leone et al., 2011; King et al., 2018). However, the current sample size and age range was not sufficient to include age as a continuous variable in the analysis. Future studies including a larger age-range are necessary to shed more light on differences in motor performance and practice-related plasticity across the life span. Secondly, while the current sample size was sufficient to observe significant and meaningful effects in both the behavioral and EEG data, further studies with larger samples are needed to replicate and confirm the effects reported here. Furthermore, physical activity levels of the current sample of older adults were high (Table 1). It is shown previously that physical fitness facilitates motor learning (Etnier et al., 2001). Therefore, the current results might not be generalizable to the entire population. However, despite the relatively active sample of older adults, we still found age-related deficits comparable with previous studies. Thirdly, we used fixed values for alpha and beta frequency bands in our EEG analysis. However, aging has been related to a decrease in the peak frequencies in both the alpha and beta bands (Doppelmayr et al., 1998; Ishii et al., 2017). It is, therefore, possible that more subtle age-related changes have been missed, and further research with individualized frequency bands is needed to provide greater insights into the effects of age on alpha and beta task-related power. Lastly, we only measured perceived fatigue at the end of the first testing session. Since peak fatigue levels were presumably reached after the practice period and fatigue may impact motor learning (Branscheidt et al., 2019), further research assessing subjective fatigue at multiple time-points and relating these measures to the objective EEG data is needed to shed more light on the possible confounding effects of fatigue processes on motor learning.

## Conclusion

In conclusion, both age and task difficulty affected motor skill acquisition and retention, as well as the associated neural plasticity. We found an age-related deterioration in motor performance which was more pronounced with increasing task difficulty and accompanied by altered patterns of brain activity. Furthermore, both age and task difficulty affected the approach to realize the task goal and improve on either the spatial or temporal dimensions of the mirror star tracing task. While we found no overall age-related deterioration in neural plasticity, task difficulty affected motor skill retention and neural plasticity specifically in older adults. Together, these results imply that the neural mechanisms of motor skill learning are dependent on both age and task difficulty.

## Data Availability Statement

The datasets presented in this study can be found in online repositories. The names of the repository/repositories and accession number(s) can be found at: DataverseNL: https://doi.org/10.34894/MCN6XM.

## Ethics Statement

The studies involving human participants were reviewed and approved by Ethische Commissie Bewegingswetenschappen, Department of Human Movement Sciences, University Medical Center Groningen, University of Groningen, Groningen, The Netherlands. The patients/participants provided their written informed consent to participate in this study.

## Author Contributions

JB, SC, and TH contributed to the design of the experiment. JB collected and analyzed the data. JB, NM, and MV prepared the analysis. JB, SC, and TH wrote the first draft of the manuscript. MV, NM, and JR revised the manuscript. All authors approved the final version of the manuscript.

## Conflict of Interest

The authors declare that the research was conducted in the absence of any commercial or financial relationships that could be construed as a potential conflict of interest.
