# Supplementary material for: Neural Correlates of Motor Skill Learning Are Dependent on Both Age and Task Difficulty
Source: Front Aging Neurosci. 2021 Mar 22;13:643132. doi: 10.3389/fnagi.2021.643132 (PMC8019720; doi:10.3389/fnagi.2021.643132)
Supplement: Supplementary file 1 [file Table_1.DOCX]

Supplementary Material


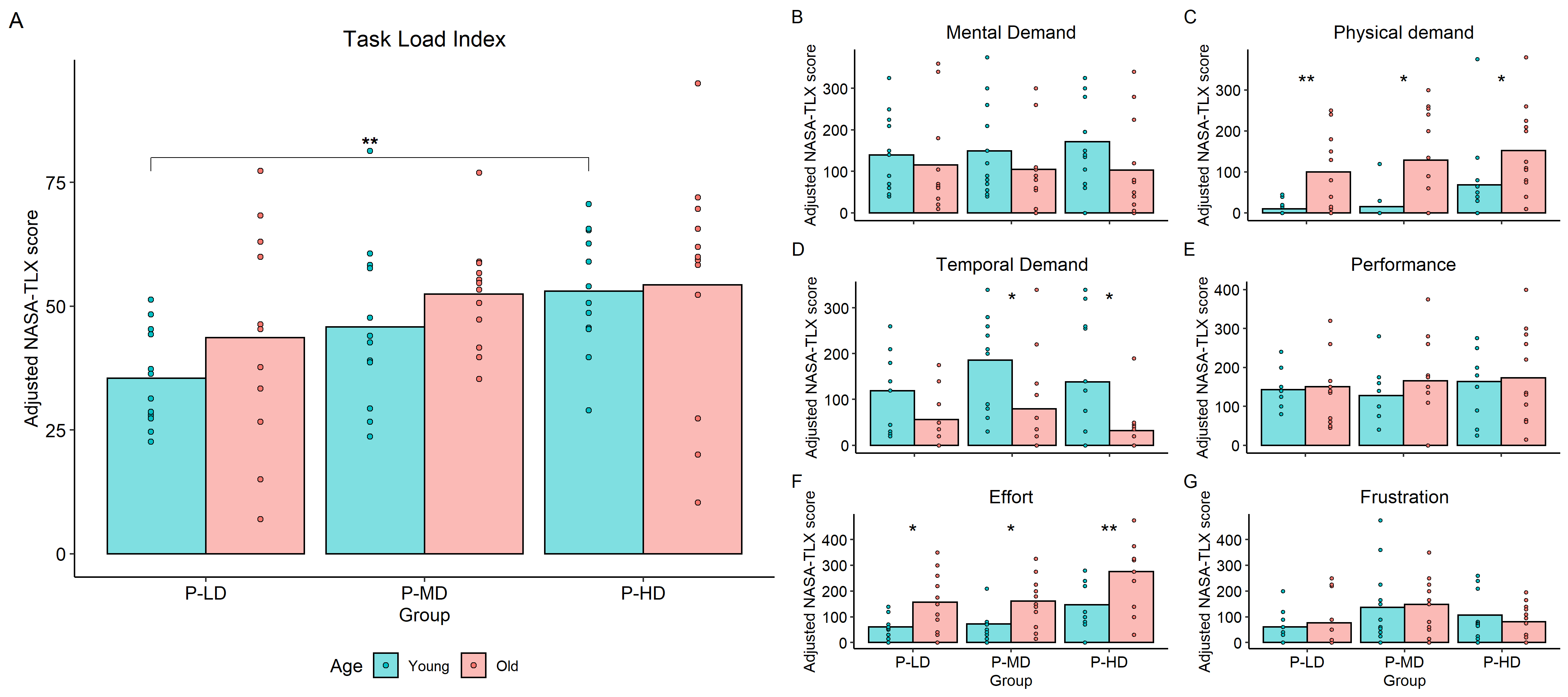


**Figure S1.** Perceived mental workload as measured by the NASA-tlx for younger (blue, left) and older (pink, right) adults for the different difficulty groups. **A)** Overall adjusted task load index, **B-G)** Adjusted ratings of the different subscales. Adjusted ratings were obtained by multiplying the given rating on a scale from 0 – 100 with the weight a participant gave to that subscale (0-5; Hart and Staveland 1988)). Dots represent individual participants. *p<0.05, **p<0.01.

| **Table S1.** Between-groups differences in mean ratings on the NASA-TLX | | | | | | | | | | | | | |
| --- | --- | --- | --- | --- | --- | --- | --- | --- | --- | --- | --- | --- | --- |
|  | **Young** | | | | | **Old** | | | | | **Age effect** | | |
|  | **P-LD** | **P-MD** | **P-HD** | **Χ^2^ (df)** | **p^a^** | **P-LD** | **P-MD** | **P-HD** | **Χ^2^ (df)** | **p^a^** | **Χ^2^ (df)** | **p** |  |
| Mental Demand | 139.6 ± 93.5 | 149.6 ± 111.5 | 171.7 ± 108 | 0.72 (2) | 0.82 | 115 ± 125.7 | 105 ± 89.5 | 102.9 ± 116.2 | 0.52 (2) | 0.85 | 4.78 (1) | 0.09 |  |
| Physical Demand | 10 ± 16.6 | 15 ± 35 | 68.7 ± 104.6 | 7.21 (2) | 0.09 | 99.5 ± 55.9 | 128.3 ± 117.7 | 151.7 ± 105.8 | 1.24 (2) | 0.71 | **19.27 (1)** | **<0.001** |  |
| Temporal Demand | 119.2 ± 92.5 | 185.8 ± 97.9 | 138.3 ± 126.5 | 2.42 (2) | 0.54 | 55.9 ± 56.74 | 79.6 ± 106.5 | 31.7 ± 53.8 | 2.10 (2) | 0.56 | **14.26 (1)** | **0.002** |  |
| Performance | 142.9 ± 50.5 | 127.5 ± 61.8 | 163.3 ± 77.3 | 2.32 (2) | 0.54 | 150.4 ± 94.4 | 165.8 ± 106.8 | 173.3 ± 117.5 | 0.28 (2) | 0.91 | 0.29 (1) | 0.73 |  |
| Effort | 60.4 ± 48.7 | 72.1 ± 69.5 | 146.7 ± 89.1 | 8.15 (2) | 0.071 | 156.8 ± 115.7 | 160.4 ± 93.9 | 275.4 ± 127.8 | 6.47 (2) | 0.10 | **13.45 (1)** | **0.002** |  |
| Frustration | 60 ± 58.8 | 137.1 ± 149.9 | 106.7 ± 101.1 | 1.53 (2) | 0.64 | 76.8 ± 103.6 | 147.5 ± 107.7 | 80 ± 69.4 | 4.06 (2) | 0.27 | 0.006 (1) | 0.94 |  |
| TLX | 35.5 ± 9.8 | 45.8 ± 16.6 | 53 ± 12.2 | **9.85 (2)** | **0.04** | 43.6 ± 22.3 | 52.4 ± 10.9 | 54.3 ± 23.9 | 1.89 (2) | 0.58 | 2.61 (1) | 0.26 |  |
| The range of each subscale is 0 – 100. Values are presented as mean ± standard deviation. *TLX*: Task Load Index, *P-LD:* Practice with Low-Difficulty Task, *P-MD:* Practice with Medium-Difficulty Task, *P-HD:* Practice with High-Difficulty Task  ^a^ FDR-adjusted p-value | | | | | | | | | | | | | |

| **Table S2**. Estimated coefficients of the multilevel models for the behavioral data | | | | | | |
| --- | --- | --- | --- | --- | --- | --- |
|  | **Movement Time** | | | **Bandwidth Error** | | |
|  | β | SE | t | β | SE | t |
| **Baseline** |  |  |  |  |  |  |
| **Fixed part** |  |  |  |  |  |  |
| Intercept | 2.70 | 0.10 | 26.62 | 17.45 | 2.88 | 6.06 |
| *Main effects* |  |  |  |  |  |  |
| Block 2^a^ | -0.24 | 0.03 | -6.86 | -6.58 | 1.09 | -6.06 |
| Block 3^a^ | -0.35 | 0.03 | -10.26 | -7.10 | 1.07 | -6.61 |
| MD^b^ | 0.11 | 0.03 | 3.24 | 8.49 | 1.07 | 7.91 |
| HD^b^ | 0.28 | 0.03 | 7.97 | 22.87 | 1.09 | 21.07 |
| P-MD^c^ | -0.006 | 0.03 | -0.049 | -5.47 | 3.36 | -1.63 |
| P-HD^c^ | 0.27 | 0.12 | 2.26 | -0.93 | 3.37 | -0.28 |
| Old^d^ | 0.73 | 0.11 | 6.86 | 11.80 | 3.05 | 3.86 |
| *Two-way interactions* |  |  |  |  |  |  |
| Block 2 x Old^a,d^ | -0.17 | 0.05 | -3.38 | 1.43 | 1.56 | 0.92 |
| Block 3 x Old^a,d^ | -0.21 | 0.05 | -4.29 | -0.82 | 1.55 | -0.53 |
| MD x Old^b,d^ | -0.07 | 0.05 | -1.49 | 3.36 | 1.55 | 2.16 |
| HD x Old^b,d^ | -0.14 | 0.05 | -2.82 | 4.33 | 1.57 | 2.76 |
| **Random Part** |  |  |  |  |  |  |
| Variance subjects | 0.16 | 0.40 |  | 128.81 | 11.35 |  |
| Variance RM | 0.02 | 0.14 |  | 20.31 | 4.51 |  |
| **Learning** |  |  |  |  |  |  |
| **Fixed Part** |  |  |  |  |  |  |
| Intercept | 2.10 | 0.11 | 19.21 | 2.25 | 0.19 | 11.69 |
| *Main effects* |  |  |  |  |  |  |
| Post^e^ | -0.37 | 0.071 | -5.24 | -0.14 | 0.13 | -1.12 |
| Retention^e^ | -0.37 | 0.071 | -5.26 | 0.31 | 0.13 | 2.39 |
| Old^d^ | 0.72 | 0.15 | 4.68 | 0.19 | 0.27 | 0.72 |
| P-MD^c^ | 0.35 | 0.15 | 2.30 | -0.14 | 0.27 | -0.52 |
| P-HD^c^ | 0.88 | 0.15 | 5.71 | 0.93 | 0.27 | 3.41 |
| *Two-way interactions* |  |  |  |  |  |  |
| Post x Old^d,e^ | -0.012 | 0.10 | -0.12 | -0.02 | 0.18 | -0.11 |
| Retention x Old^d,e^ | 0.25 | 0.10 | 2.52 | -0.67 | 0.18 | -3.73 |
| Post x P-MD^c,e^ | 0.27 | 0.10 | 2.66 | -0.41 | 0.18 | -2.21 |
| Retention x P-MD^c,e^ | 0.17 | 0.10 | 1.72 | -0.71 | 0.18 | -3.84 |
| Post x P-HD^c,e^ | 0.055 | 0.10 | 0.55 | -0.066 | 0.18 | -0.37 |
| Retention x P-HD^c,e^ | -0.042 | 0.10 | -0.43 | -0.37 | 0.18 | -2.00 |
| Old x P-MD^c,d^ | -0.36 | 0.22 | -1.66 | 0.83 | 0.38 | 2.17 |
| Old x P-HD^c,d^ | -0.55 | 0.22 | -2.51 | 0.50 | 0.38 | 1.31 |
| *Three-way interactions* |  |  |  |  |  |  |
| Post x Old x P-MD^c,d,e^ | -0.074 | 0.14 | -0.53 | -0.03 | 0.26 | -0.11 |
| Retention x Old x P-MD^c,d,e^ | -0.041 | 0.14 | -0.29 | 0.69 | 0.26 | 2.67 |
| Post x Old x P-HD^c,d,e^ | 0.094 | 0.14 | 0.66 | 0.035 | 0.26 | 0.13 |
| Retention x Old x P-HD^c,d,e^ | 0.054 | 0.14 | 0.38 | 0.60 | 0.26 | 2.33 |
| **Random Part** |  |  |  |  |  |  |
| Variance subjects | 0.11 | 0.34 |  | 0.35 | 0.59 |  |
| Variance RM | 0.03 | 0.17 |  | 0.098 | 0.31 |  |
| Reference categories: ^a^Block 1, ^b^LD, ^c^P-LD, ^d^Young, ^e^Pre | | | | | | |

| **Table S3.** Estimated coefficients of the multilevel models for the TR-Power data | | | | | | | | | | | | | | | | | | |  |
| --- | --- | --- | --- | --- | --- | --- | --- | --- | --- | --- | --- | --- | --- | --- | --- | --- | --- | --- | --- |
| **Baseline** | **Frontal** | | | | **Motor** | | | | | | | | **Parietal** | | | | | |  |
|  | β | SE | t | | β | SE | | | t | | | | β | | SE | | t |  |  |
| **Alpha band** |  |  |  | |  |  | | |  | | | |  | |  | |  |  |  |
| **Fixed Part** |  |  |  | |  |  | | |  | | | |  | |  | |  |  |  |
| Intercept | 4.13 | 0.11 | 36.81 | | 4.07 | 0.12 | | | 33.9 | | | | 3.95 | | 0.14 | | 29.1 |  |  |
| *Main effects* |  |  |  | |  |  | | |  | | | |  | |  | |  |  |  |
| Block 2^a^ | -0.011 | 0.021 | -0.52 | | -0.005 | 0.02 | | | -0.21 | | | | 0.014 | | 0.026 | | 0.56 |  |  |
| Block 3^a^ | 0.011 | 0.021 | 0.55 | | 0.019 | 0.02 | | | 0.81 | | | | 0.020 | | 0.025 | | 0.79 |  |  |
| MD^b^ | 0.026 | 0.030 | 0.87 | | -0.031 | 0.033 | | | -0.95 | | | | -0.031 | | 0.036 | | -0.86 |  |  |
| HD^b^ | 0.025 | 0.030 | 0.83 | | -0.005 | 0.033 | | | -0.14 | | | | -0.0067 | | 0.036 | | -0.19 |  |  |
| Ipsilateral^c^ | 0.057 | 0.030 | 1.89 | | 0.039 | 0.033 | | | 1.18 | | | | -0.072 | | 0.036 | | -1.97 |  |  |
| P-MD^d^ | -0.035 | 0.13 | -0.26 | | -0.068 | 0.14 | | | -0.48 | | | | -0.11 | | 0.16 | | -0.70 |  |  |
| P-HD^d^ | 0.020 | 0.13 | 0.15 | | -0.023 | 0.14 | | | -0.16 | | | | -0.03 | | 0.16 | | -0.18 |  |  |
| Old^e^ | 0.16 | 0.11 | 1.39 | | 0.21 | 0.12 | | | 1.71 | | | | 0.24 | | 0.14 | | 1.74 |  |  |
| *Two-way interactions* |  |  |  | |  |  | | |  | | | |  | |  | |  |  |  |
| Block 2 x Old^a,e^ | 0.070 | 0.030 | 2.36 | | 0.042 | 0.033 | | | 1.25 | | | | 0.0097 | | 0.035 | | 0.27 |  |  |
| Block 3 x Old^a,e^ | 0.045 | 0.029 | 1.54 | | 0.036 | 0.033 | | | 1.09 | | | | 0.041 | | 0.035 | | 1.19 |  |  |
| MD x Old^b,e^ | -0.015 | 0.042 | -0.36 | | 0.014 | 0.047 | | | 0.30 | | | | 0.014 | | 0.049 | | 0.29 |  |  |
| HD x Old^b,e^ | -0.025 | 0.042 | -0.60 | | 0.023 | 0.047 | | | 0.50 | | | | -0.013 | | 0.049 | | -0.27 |  |  |
| Ipsilateral x Old^c,e^ | -0.062 | 0.042 | -1.48 | | -0.050 | 0.047 | | | -1.06 | | | | 0.13 | | 0.05 | | 2.65 |  |  |
| MD x Ipsilateral^d,e^ | 0.0024 | 0.042 | 0.057 | | 0.058 | 0.046 | | | 1.24 | | | | 0.11 | | 0.05 | | 2.11 |  |  |
| HD x Ipsilateral^d,e^ | 0.0031 | 0.042 | 0.075 | | 0.099 | 0.047 | | | 2.12 | | | | 0.10 | | 0.05 | | 2.04 |  |  |
| *Three-way interactions* |  |  |  | |  |  | | |  | | | |  | |  | |  |  |  |
| MD x Ipsilateral x Old^c,d,e^ | -0.027 | 0.059 | -0.45 | | -0.073 | 0.066 | | | -1.10 | | | | -0.095 | | 0.071 | | -1.35 |  |  |
| HD x Ipsilateral x Old^c,d,e^ | 0.0002 | 0.059 | 0.004 | | -0.14 | 0.066 | | | -2.05 | | | | -0.13 | | 0.070 | | -1.83 |  |  |
| **Random Part** |  |  |  | |  |  | | |  | | | |  | |  | |  |  |  |
| Variance subjects | 0.21 | 0.46 |  | | 0.24 | 0.50 | | |  | | | | 0.31 | | 0.56 | |  |  |  |
| Variance RM | 0.01 | 0.12 |  | | 0.02 | 0.13 | | |  | | | | 0.02 | | 0.14 | |  |  |  |
|  |  |  |  | |  |  | | |  | | |  | | |  | |  | | |
| **Beta band** |  |  |  | |  |  | | |  | | |  | | |  | |  | | |
| **Fixed Part** |  |  |  | |  |  | | |  | | |  | | |  | |  | | |
| Intercept | 4.66 | 0.10 | 45.1 | | 4.47 | 0.11 | | | 41.6 | | | 4.45 | | | 0.098 | | 45.5 | | |
| *Main effects* |  |  |  | |  |  | | |  | | |  | | |  | |  | | |
| Block 2^a^ | 0.033 | 0.028 | 1.16 | | 0.019 | 0.028 | | | 0.67 | | | 0.028 | | | 0.023 | | 1.20 | | |
| Block 3^a^ | 0.00741 | 0.028 | 0.26 | | 0.013 | 0.028 | | | 0.49 | | | 0.0098 | | | 0.023 | | 0.43 | | |
| MD^b^ | -0.011 | 0.040 | -0.28 | | -0.021 | 0.039 | | | -0.54 | | | -0.036 | | | 0.033 | | -1.10 | | |
| HD^b^ | -0.068 | 0.040 | -1.69 | | -0.035 | 0.040 | | | -0.87 | | | -0.009 | | | 0.033 | | -0.28 | | |
| Ipsilateral^c^ | -0.027 | 0.040 | -0.67 | | -0.0071 | 0.040 | | | -0.18 | | | -0.023 | | | 0.033 | | -0.71 | | |
| P-MD^d^ | -0.22 | 0.12 | -1.79 | | -0.19 | 0.13 | | | -0.52 | | | -0.087 | | | 0.12 | | -0.75 | | |
| P-HD^d^ | -0.13 | 0.12 | -1.07 | | -0.11 | 0.13 | | | -0.87 | | | 0.0024 | | | 0.12 | | 0.021 | | |
| Old^e^ | -0.002 | 0.11 | -0.015 | | -0.078 | 0.11 | | | -0.69 | | | -0.099 | | | 0.10 | | -0.98 | | |
| *Two-way interactions* |  |  |  | |  |  | | |  | | |  | | |  | |  | | |
| Block 2 x Old^a,e^ | -0.031 | 0.04 | -0.76 | | 0.026 | 0.040 | | | 0.65 | | | -0.01 | | | 0.033 | | -0.31 | | |
| Block 3 x Old^a,e^ | 0.054 | 0.040 | 1.37 | | -0.015 | 0.040 | | | -0.37 | | | 0.0069 | | | 0.033 | | 0.21 | | |
| MD x Old^b,e^ | 0.019 | 0.057 | 0.34 | | 0.0097 | 0.057 | | | 0.17 | | | 0.035 | | | 0.046 | | 0.76 | | |
| HD x Old^b,e^ | -0.033 | 0.057 | -0.59 | | 0.031 | 0.058 | | | 0.54 | | | -0.030 | | | 0.047 | | -0.64 | | |
| Ipsilateral x Old^c,e^ | 0.020 | 0.057 | 0.34 | | -0.0079 | 0.057 | | | -0.14 | | | 0.044 | | | 0.047 | | 0.94 | | |
| MD x Ipsilateral^d,e^ | -0.00015 | 0.057 | -0.003 | | -0.0088 | 0.056 | | | -0.16 | | | 0.034 | | | 0.046 | | 0.74 | | |
| HD x Ipsilateral^d,e^ | 0.055 | 0.056 | 0.97 | | 0.034 | 0.056 | | | 0.60 | | | 0.020 | | | 0.046 | | 0.44 | | |
| *Three-way interactions* |  |  |  | |  |  | | |  | | |  | | |  | |  | | |
| MD x Ipsilateral x Old^c,d,e^ | -0.016 | 0.08 | -0.20 | | -0.00023 | 0.080 | | | -0.003 | | | -0.026 | | | 0.066 | | -0.40 | | |
| HD x Ipsilateral x Old^c,d,e^ | 0.026 | 0.080 | 0.33 | | -0.063 | 0.081 | | | -0.78 | | | -0.0096 | | | 0.066 | | -0.15 | | |
| **Random Part** |  |  |  | |  |  | | |  | | |  | | |  | |  | | |
| Variance subjects | 0.17 | 0.41 |  | | 0.19 | 0.43 | | |  | | | 0.16 | | | 0.39 | |  | | |
| Variance RM | 0.027 | 0.16 |  | | 0.027 | | | 0.16 | | |  | 0.018 | | | 0.13 | |  | | |
| **Learning** | **Frontal** | | | | **Motor** | | | | | | | **Parietal** | | | | | | | |
|  | β | SE | t | | β | SE | | | t | | | β | | SE | | | t | | |
|  |  |  |  | |  |  | | |  | | |  | |  | |  | | | |
| **Alpha band** |  |  |  | |  |  | | |  | | |  | |  | | |  | | |
| **Fixed Part** |  |  |  | |  |  | | |  | | |  | |  | | |  | | |
| Intercept | -28.39 | 10.06 | -2.82 | | -29.29 | 9.49 | | | -3.09 | | | -38.15 | | 9.18 | | | -4.15 | | |
| *Main effects* |  |  |  | |  |  | | |  | | |  | |  | | |  | | |
| Post^f^ | 0.060 | 6.63 | 0.009 | | -6.45 | 6.62 | | | -0.97 | | | -7.76 | | 5.76 | | | -1.35 | | |
| Retention^f^ | 1.00 | 6.81 | 0.15 | | -2.83 | 7.003 | | | -0.41 | | | -6.56 | | 5.94 | | | -1.11 | | |
| Ipsilateral^c^ | -4.06 | 6.92 | -0.59 | | -1.48 | 6.72 | | | -0.22 | | | -6.11 | | 5.70 | | | -1.07 | | |
| Old^e^ | 7.62 | 14.06 | 0.54 | | 12.68 | 13.24 | | | 0.96 | | | 15.61 | | 12.93 | | | 1.21 | | |
| P-MD^d^ | 2.44 | 14.06 | 0.17 | | -1.14 | 13.24 | | | -0.086 | | | -4.57 | | 12.93 | | | -0.35 | | |
| P-HD^d^ | -3.77 | 14.13 | -0.27 | | -12.78 | 13.24 | | | -0.97 | | | -5.59 | | 12.93 | | | -0.43 | | |
| *Two-way interactions* |  |  |  | |  |  | | |  | | |  | |  | | |  | | |
| Post x Ipsilateral^c,f^ | 10.19 | 9.33 | 1.09 | | 7.22 | 9.18 | | | 0.79 | | | 10.71 | | 7.99 | | | 1.34 | | |
| Retention x Ipsilateral^c,f^ | 12.16 | 9.52 | 1.28 | | 5.92 | 9.65 | | | 0.61 | | | 13.86 | | 8.18 | | | 1.70 | | |
| Post x Old^e,f^ | -14.57 | 9.11 | -1.60 | | -16.55 | 9.11 | | | -1.82 | | | -16.96 | | 7.93 | | | -2.14 | | |
| Retention x Old^e,f^ | 8.08 | 9.50 | 0.85 | | 2.33 | 9.65 | | | 0.24 | | | 4.64 | | 8.30 | | | 0.56 | | |
| Post x P-MD^d,f^ | -10.77 | 9.37 | -1.15 | | -8.98 | 9.37 | | | -0.96 | | | -6.02 | | 8.04 | | | -0.75 | | |
| Retention x P-MD^d,f^ | -0.38 | 9.36 | -0.041 | | -3.67 | 9.50 | | | -0.39 | | | 12.70 | | 8.06 | | | 1.58 | | |
| Post x P-HD^d,f^ | -1.87 | 9.23 | -0.20 | | 2.91 | 9.22 | | | 0.32 | | | -3.78 | | 8.04 | | | -0.47 | | |
| Retention x P-HD^d,f^ | 0.91 | 9.36 | 0.097 | | 5.32 | 9.39 | | | 0.57 | | | 0.03 | | 8.17 | | | 0.004 | | |
| Ipsilateral x Old^c,e^ | -1.12 | 9.33 | -0.12 | | -10.76 | 9.18 | | | -1.17 | | | 3.67 | | 8.01 | | | 0.46 | | |
| Ipsilateral x P-MD^c,d^ | 4.01 | 9.33 | 0.43 | | 0.34 | 9.18 | | | 0.037 | | | 12.50 | | 7.89 | | | 1.58 | | |
| Ipsilateral x P-HD^c,d^ | 8.15 | 9.45 | 0.86 | | 11.86 | 9.18 | | | 1.29 | | | 4.82 | | 8.00 | | | 0.60 | | |
| Old x P-MD^d,e^ | -9.38 | 19.76 | -0.48 | | -14.92 | 18.60 | | | -0.80 | | | -6.44 | | 18.23 | | | -0.35 | | |
| Old x P-HD^d,e^ | 10.34 | 19.94 | 0.52 | | 11.55 | 18.74 | | | 0.62 | | | 3.76 | | 18.34 | | | 0.21 | | |
| *Three-way interactions* |  |  |  | |  |  | | |  | | |  | |  | | |  | | |
| Post x Ipsilateral x Old^c,e,f^ | -3.74 | 12.86 | -0.29 | | 2.99 | 12.84 | | | 0.23 | | | -2.36 | | 11.23 | | | -0.21 | | |
| Retention x Ipsilateral x Old^c,e,f^ | -17.61 | 13.30 | -1.32 | | -3.69 | 13.38 | | | -0.28 | | | -6.17 | | 11.59 | | | -0.53 | | |
| Post x Ipsilateral x P-MD^c,e,f^ | -4.11 | 13.20 | -0.31 | | 4.04 | 12.99 | | | 0.31 | | | -11.07 | | 11.23 | | | -0.99 | | |
| Retention x Ipsilateral x P-MD^c,e,f^ | -4.81 | 13.08 | -0.37 | | 21.89 | 13.25 | | | 1.65 | | | -17.78 | | 11.32 | | | -1.58 | | |
| Post x Ipsilateral x P-HD^c,e,f^ | -16.07 | 12.95 | -1.24 | | -12.48 | 12.92 | | | -0.97 | | | -0.61 | | 11.30 | | | -0.054 | | |
| Retention x Ipsilateral x P-HD^c,e,f^ | -12.25 | 13.20 | -0.93 | | -6.73 | 13.09 | | | -0.52 | | | -10.46 | | 11.44 | | | -0.91 | | |
| Post x Old x P-MD^d,e,f^ | 4.15 | 13.07 | 0.32 | | 10.61 | 12.96 | | | 0.82 | | | 7.21 | | 11.22 | | | 0.64 | | |
| Retention x Old x P-MD^d,e,f^ | -20.19 | 13.07 | -1.55 | | -4.88 | 13.17 | | | -0.37 | | | -28.86 | | 11.41 | | | -2.53 | | |
| Post x Old x P-HD^d,e,f^ | 16.24 | 13.13 | 1.24 | | 12.82 | 13.12 | | | 0.98 | | | 18.80 | | 11.44 | | | 1.64 | | |
| Retention x Old x P-HD^d,e,f^ | -23.21 | 13.35 | -1.74 | | -8.45 | 13.42 | | | -0.63 | | | 9.65 | | 11.81 | | | 0.82 | | |
| Ipsilateral x Old x P-MD^d,e,f^ | -2.09 | 12.94 | -0.16 | | 15.58 | 12.75 | | | 1.22 | | | -9.75 | | 11.12 | | | -0.88 | | |
| Ipsilateral x Old x P-HD^d,e,f^ | -11.47 | 13.18 | -0.87 | | -9.77 | 13.05 | | | -0.75 | | | -10.73 | | 11.57 | | | -0.93 | | |
| *Four-way interactions* |  |  |  | |  |  | | |  | | |  | |  | | |  | | |
| Post x Ipsilateral x Old x P-MD^c,d,e,f^ | 3.91 | 18.41 | 0.21 | | -10.92 | 18.15 | | | -0.60 | | | 9.47 | | 15.86 | | | 0.60 | | |
| Retention x Ipsilateral x Old x P-MD^c,d,e,f^ | 14.99 | 18.44 | 0.81 | | -25.18 | 18.44 | | | -1.37 | | | 10.60 | | 16.06 | | | 0.66 | | |
| Post x Ipsilateral x Old x P-HD^c,d,e,f^ | 5.88 | 18.37 | 0.32 | | 6.57 | 18.40 | | | 0.36 | | | 6.56 | | 16.18 | | | 0.40 | | |
| Retention x Ipsilateral x Old x P-HD^c,d,e,f^ | 24.71 | 18.66 | 1.32 | | 5.45 | 18.67 | | | 0.29 | | | -12.85 | | 16.67 | | | -0.77 | | |
| **Random Part** |  |  |  | |  |  | | |  | | |  | |  | | |  | | |
| Variance subjects | 922.2 | 30.37 |  | | 788.9 | 28.09 | | |  | | | 813.8 | | 28.53 | | |  | | |
| Variance RM | 235 | 15.33 |  | | 234.7 | 15.32 | | |  | | | 178.6 | | 13.37 | | |  | | |
| **Beta band** |  |  |  |  | | |  | | |  | | |  | |  | |  |  |  |
| **Fixed Part** |  |  |  |  | | |  | | |  | | |  | |  | |  |  |  |
| Intercept | 4.62 | 0.12 | 38.54 | 4.40 | | | 0.12 | | | 35.34 | | | 4.46 | | 0.12 | | 38.4 |  |  |
| *Main effects* |  |  |  |  | | |  | | |  | | |  | |  | |  |  |  |
| Post^f^ | -0.21 | 0.078 | -2.74 | -0.12 | | | 0.077 | | | -1.63 | | | -0.090 | | 0.070 | | -1.28 |  |  |
| Retention^f^ | -0.18 | 0.079 | -2.24 | -0.11 | | | 0.079 | | | -1.42 | | | 0.0071 | | 0.074 | | 0.095 |  |  |
| Ipsilateral^c^ | -0.11 | 0.078 | -1.42 | -0.018 | | | 0.078 | | | -0.23 | | | -0.094 | | 0.071 | | -1.32 |  |  |
| Old^e^ | 0.14 | 0.17 | 0.83 | 0.10 | | | 0.18 | | | 0.57 | | | -0.031 | | 0.17 | | -0.19 |  |  |
| P-MD^d^ | -0.05 | 0.17 | -0.31 | -0.039 | | | 0.17 | | | -0.22 | | | -0.062 | | 0.16 | | -0.38 |  |  |
| P-HD^d^ | -0.17 | 0.17 | -0.99 | -0.048 | | | 0.18 | | | -0.27 | | | 0.011 | | 0.16 | | 0.065 |  |  |
| *Two-way interactions* |  |  |  |  | | |  | | |  | | |  | |  | |  |  |  |
| Post x Ipsilateral^c,f^ | 0.16 | 0.11 | 1.46 | 0.082 | | | 0.11 | | | 0.75 | | | 0.060 | | 0.099 | | 0.61 |  |  |
| Retention x Ipsilateral^c,f^ | 0.25 | 0.11 | 2.26 | 0.16 | | | 0.11 | | | 1.47 | | | 0.16 | | 0.10 | | 1.52 |  |  |
| Post x Old^e,f^ | -0.15 | 0.11 | -1.34 | -0.29 | | | 0.11 | | | -2.62 | | | -0.27 | | 0.10 | | -2.62 |  |  |
| Retention x Old^e,f^ | -0.01 | 0.12 | -0.10 | -0.02 | | | 0.12 | | | -0.19 | | | -0.043 | | 0.11 | | -0.38 |  |  |
| Post x P-MD^d,f^ | 0.036 | 0.11 | 0.32 | -0.07 | | | 0.11 | | | -0.63 | | | -0.0032 | | 0.10 | | -0.032 |  |  |
| Retention x P-MD^d,f^ | 0.12 | 0.11 | 1.05 | 0.091 | | | 0.11 | | | 0.84 | | | 0.13 | | 0.10 | | 1.30 |  |  |
| Post x P-HD^d,f^ | 0.32 | 0.11 | 2.89 | 0.052 | | | 0.11 | | | 0.47 | | | -0.022 | | 0.099 | | -0.22 |  |  |
| Retention x P-HD^d,f^ | 0.30 | 0.11 | 2.69 | 0.23 | | | 0.11 | | | 2.04 | | | 0.055 | | 0.10 | | 0.54 |  |  |
| Ipsilateral x Old^c,e^ | 0.04 | 0.11 | 0.38 | -0.24 | | | 0.11 | | | -2.18 | | | 0.0065 | | 0.11 | | 0.060 |  |  |
| Ipsilateral x P-MD^c,d^ | -0.006 | 0.11 | -0.058 | -0.031 | | | 0.11 | | | -0.29 | | | 0.15 | | 0.099 | | 1.51 |  |  |
| Ipsilateral x P-HD^c,d^ | 0.21 | 0.11 | 1.92 | 0.017 | | | 0.11 | | | 0.16 | | | 0.10 | | 0.10 | | 1.038 |  |  |
| Old x P-MD^d,e^ | -0.31 | 0.24 | -1.30 | -0.33 | | | 0.25 | | | -1.35 | | | -0.16 | | 0.23 | | -0.69 |  |  |
| Old x P-HD^d,e^ | -0.22 | 0.24 | -0.90 | -0.25 | | | 0.25 | | | -1.004 | | | -0.071 | | 0.23 | | -0.30 |  |  |
| *Three-way interactions* |  |  |  |  | | |  | | |  | | |  | |  | |  |  |  |
| Post x Ipsilateral x Old^c,e,f^ | 0.082 | 0.15 | 0.53 | 0.18 | | | 0.16 | | | 1.14 | | 0.30 | | | 0.15 | | 2.003 | | |
| Retention x Ipsilateral x Old^c,e,f^ | -0.17 | 0.16 | -1.055 | 0.0045 | | | 0.16 | | | 0.028 | | -0.048 | | | 0.16 | | -0.31 | | |
| Post x Ipsilateral x P-MD^c,e,f^ | -0.073 | 0.16 | -0.47 | 0.041 | | | 0.16 | | | 0.16 | | -0.11 | | | 0.14 | | -0.78 | | |
| Retention x Ipsilateral x P-MD^c,e,f^ | -0.14 | 0.16 | -0.91 | 0.069 | | | 0.15 | | | 0.44 | | -0.24 | | | 0.14 | | -1.67 | | |
| Post x Ipsilateral x P-HD^c,e,f^ | -0.29 | 0.15 | -1.88 | -0.028 | | | 0.16 | | | -0.18 | | 0.059 | | | 0.14 | | 0.42 | | |
| Retention x Ipsilateral x P-HD^c,e,f^ | -0.37 | 0.16 | -2.37 | -0.18 | | | 0.16 | | | -1.12 | | -0.17 | | | 0.14 | | -1.17 | | |
| Post x Old x P-MD^d,e,f^ | 0.18 | 0.16 | 1.13 | 0.23 | | | 0.16 | | | 1.47 | | 0.17 | | | 0.14 | | 1.18 | | |
| Retention x Old x P-MD^d,e,f^ | -0.017 | 0.16 | -0.11 | 0.019 | | | 0.16 | | | 0.12 | | -0.11 | | | 0.15 | | -0.76 | | |
| Post x Old x P-HD^d,e,f^ | 0.072 | 0.16 | 0.46 | 0.30 | | | 0.16 | | | 1.87 | | 0.26 | | | 0.14 | | 1.76 | | |
| Retention x Old x P-HD^d,e,f^ | 0.013 | 0.16 | 0.079 | 0.10 | | | 0.16 | | | 0.64 | | 0.072 | | | 0.15 | | 0.47 | | |
| Ipsilateral x Old x P-MD^d,e,f^ | 0.034 | 0.16 | 0.21 | 0.23 | | | 0.16 | | | 1.45 | | -0.031 | | | 0.15 | | -0.21 | | |
| Ipsilateral x Old x P-HD^d,e,f^ | -0.077 | 0.16 | -0.50 | 0.27 | | | 0.15 | | | 1.73 | | -0.073 | | | 0.15 | | -0.49 | | |
| *Four-way interactions* |  |  |  |  | | |  | | |  | |  | | |  | |  | | |
| Post x Ipsilateral x Old x P-MD^c,d,e,f^ | -0.14 | 0.22 | -0.64 | -0.13 | | | 0.22 | | | -0.57 | | -0.23 | | | 0.21 | | -1.12 | | |
| Retention x Ipsilateral x Old x P-MD^c,d,e,f^ | 0.16 | 0.23 | 0.71 | -0.14 | | | 0.22 | | | -0.65 | | 0.074 | | | 0.21 | | 0.35 | | |
| Post x Ipsilateral x Old x P-HD^c,d,e,f^ | -0.013 | 0.22 | -0.059 | -0.24 | | | 0.22 | | | -1.065 | | -0.30 | | | 0.20 | | -1.47 | | |
| Retention x Ipsilateral x Old x P-HD^c,d,e,f^ | 0.20 | 0.22 | 0.89 | -0.12 | | | 0.22 | | | -0.51 | | 0.029 | | | 0.21 | | 0.14 | | |
| **Random Part** |  |  |  |  | | |  | | |  | |  | | |  | |  | | |
| Variance subjects | 0.13 | 0.37 |  | 0.15 | | | 0.39 | | |  | | 0.13 | | | 0.36 | |  | | |
| Variance RM | 0.034 | 0.18 |  | 0.034 | | | 0.18 | | |  | | 0.028 | | | 0.17 | |  | | |
| Reference groups: ^a^Block 1, ^b^LD, ^c^Contralateral, ^d^P-LD, ^e^Young, ^f^Pre | | | | | | | | | | | | | | | | | | | |
